# Recreational physical activity and health-related quality of life among breast cancer survivors: a systematic review

**DOI:** 10.1007/s11136-025-03992-1

**Published:** 2025-07-02

**Authors:** Emma Tian, Oliver W. A. Wilson, Kaitlyn M. Wojcik, Gisela Butera, Jacob Schneider, Laura Q. Rogers, Wendy Demark-Wahnefried, Jinani Jayasekera

**Affiliations:** 1 Division of Intramural Research at the National Institute on Minority Health and Health Disparities, National Institutes of Health, Bethesda, MD USA; 2 Division of General Internal Medicine and Population Science, Department of Medicine, Heersink School of Medicine at University of Alabama at Birmingham, Birmingham, AL USA; 3 Department of Nutrition Sciences, School of Public Health at University of Alabama at Birmingham, Birmingham, AL USA; 4 Office of Research Services, National Institutes of Health Library, Bethesda, MD USA

**Keywords:** Breast cancer patients, Exercise, Patient reported outcomes, Well-being

## Abstract

**Purpose:**

Breast cancer survivors are at increased risk of poor health-related quality of life (HRQOL). Clinical trials suggest physical activity interventions may improve HRQOL, however, evidence on whether these benefits extend to physical activity in real-world settings is limited. We aimed to evaluate the association between post-diagnosis recreational physical activity and HRQOL among breast cancer survivors in the observational literature and compare findings with clinical trial data.

**Methods:**

A systematic review was conducted, searching seven databases for studies published between January 2003 and October 2024. Study characteristics and adjusted analyses of the association between recreational physical activity and HRQOL were extracted. A qualitative synthesis categorized HRQOL outcomes into global, physical, emotional, social, and breast cancer-specific domains. Risk of bias was assessed, and findings from observational studies were compared with those from clinical trials.

**Results:**

The search identified 5831 sources, with 11 studies meeting inclusion criteria. For most domains, studies reported positive associations between aerobic activity and HRQOL. Meeting aerobic activity guidelines was found to have clinically meaningful positive associations with global HRQOL (Five studies), physical HRQOL (Two studies), and breast cancer-specific HRQOL (One study). Fewer studies reported on emotional/social domains of HRQOL or the association between muscle-strengthening activity and HRQOL. Overall, observational findings were consistent with clinical trial findings.

**Conclusions:**

Observational evidence suggests that aerobic activity guideline adherence may be associated with increased global and physical HRQOL. These findings indicate that benefits observed in trials may extend to real-world recreational aerobic activity. Further research is needed on muscle-strengthening activity and activity dosage.

**Supplementary Information:**

The online version contains supplementary material available at 10.1007/s11136-025-03992-1.

## Plain English Summary

Clinical trials have shown that physical activity interventions can improve breast cancer survivors’ quality of life. However, it is unclear whether quality of life improvements extend to everyday recreational physical activity in the broader population of breast cancer survivors. To fill this gap in the literature, we examined the relationship between physical activity and health-related quality of life in real-world observational studies. The main question was whether participation in recreational physical activity (e.g., walking, biking, swimming) can improve the well-being of breast cancer survivors.

We found that breast cancer survivors engaging in at least 150 min of moderate intensity aerobic activity showed greater overall quality of life. These results were similar to those observed in clinical trials, suggesting that the benefits of physical activity extend to a broad range of breast cancer survivors, beyond those typically included in clinical trials. However, further research is needed to explore the effects of other types of exercise, such as strength training (e.g., squats) on quality of life. These findings highlight the potential for recreational aerobic physical activity to enhance the health and well-being of breast cancer survivors.

## Introduction

Breast cancer is the most frequently diagnosed cancer in women (2.3 million new cases in 2022 [1]), and incidence rates continue to increase worldwide [2]. Meanwhile, the current five-year relative survival rate for breast cancer in the U.S. is 91% across all stages combined [3]. Thus, there is a large population of breast cancer survivors whose health-related quality of life (HRQOL) warrants attention. HRQOL is a multidimensional assessment of how disease and treatment affect a patient’s sense of overall function and well-being [4]. It encompasses several domains, including physical (e.g., fatigue), emotional (e.g., sadness), social (e.g., companionship), disease-specific concerns (e.g., hair loss), and global (overall) HRQOL [5–7]. Breast cancer survivors experience significant declines in HRQOL during diagnosis and treatment [8, 9] that can persist into long-term survivorship [10]. Studies show that breast cancer survivors are more likely to report pain, fatigue, and feelings of sadness compared to their peers without a cancer history [10, 11]. Physical activity has been identified as a promising, low-risk behavior that may address poor HRQOL among breast cancer survivors [12–14].

Exercise guidelines endorsed by the American Cancer Society, American College of Sports Medicine, and American Society of Clinical Oncology recommend regular aerobic and resistance activity to cancer survivors [15–17], informed in part by clinical trials of physical activity showing improvements in HRQOL among survivors [13, 18, 19]. For example, meta-analyses have shown that breast cancer survivors randomized to a physical activity intervention experience increased global HRQOL compared to those assigned to a control arm (e.g., usual care) [13, 18, 19]. However, it is estimated that only 37.7% of U.S. breast cancer survivors meet aerobic activity guidelines, while just 17.6% adhere to muscle-strengthening guidelines [20].

Randomized controlled trials are considered the gold standard to evaluate interventions’ efficacy [21]. Efficacy could quantify how well a physical activity intervention will work under controlled and ideal conditions of a clinical trial [22]. For instance, clinical trials may screen for high performance status, low comorbidities, and/or high readiness to engage in physical activity, which may not reflect women in real-world practice [23, 24]. Moreover, clinical trials may involve resource-intensive supervised and/or specialized individual or group sessions, which may not be accessible to breast cancer survivors outside of a clinical trial [25–28]. Given the select, controlled conditions of trials, it is unclear whether the benefits associated with interventions found in trials will extend to real-world, habitual physical activity.

In contrast, effectiveness refers to the impact of physical activity in real-world and routine practice, where factors such as patient heterogeneity (e.g., comorbidities), variations in adherence to physical activity, and broader conditions (e.g., access) are at play. While clinical trials are critical for establishing the efficacy of physical activity, observational studies are necessary to assess the effectiveness of physical activity in broader, more diverse populations [29, 30]. Observational studies may also measure habitual activity, rather than prompting activity through intervention as seen in trials. This observed activity may be more indicative of real-world engagement and offer insight into potential improvements in HRQOL that can be achieved and sustained outside of the context of an intervention. To date, no studies have directly compared physical activity-HRQOL associations between observational and trial data. Therefore, the overarching goal of our study was to summarize the effectiveness of recreational physical activity on HRQOL among breast cancer survivors.

The primary aim of this systematic review was to assess and critically evaluate the observational literature on the association between post-diagnosis recreational/leisure-time physical activity and HRQOL domains (i.e., physical, emotional, social, disease-specific concerns, and global) among breast cancer survivors. A secondary aim was to summarize the potential differences in findings across observational studies and clinical trials.

## Methods

### Data sources and search strategy

This systematic review followed the Preferred Reporting Items for Systematic Reviews and Meta-Analyses (PRISMA) guidelines [31] and was registered in Open Science Framework (osf.io/9zua5). Seven databases were searched from January 2003 to October 2024 (MEDLINE via PubMed, PsycInfo, Embase, Scopus, Web of Science, Cochrane CENTRAL, and CINAHL Plus). We identified empirical English-language observational studies on the association between recreational physical activity and HRQOL in breast cancer survivors. A trained librarian (GB) designed the search strategy using terms such as “Breast Neoplasms,” “Exercise,” and “quality-of-life” (see Supplement 1). We also conducted forward and backward citation searches of extracted sources.

### Exposure definition

Physical activity is considered any movement that expends energy [32]. The World Health Organization and U.S. Department of Health and Human Services recommend adults engage in moderate-intensity aerobic activity for at least 150 min per week and muscle-strengthening activity at least twice per week [33, 34]. Guidelines for cancer survivors recommend that survivors gradually progress towards these activity levels [17].

Physical activity can be categorized into domains such as occupational, transport, household, and recreational activities [32]. Our main exposure of interest in this review was recreational, or leisure-time, physical activity, which encompasses discretionary exercise, sports, and other leisure-time pursuits [35]. We selected recreational physical activity as the main exposure as it is the most modifiable domain of physical activity [36], is often consistently measured across the literature [32, 37], and is more closely associated with improved patient-reported outcomes, such as fatigue and depressive symptoms, likely due to greater enjoyment and intrinsic motivation [38].

### Inclusion criteria

Studies were included if they examined the relationship between recreational physical activity (aerobic and/or muscle-strengthening) and HRQOL (Supplement 2). When physical activity measures were unclear, we contacted authors for clarification. HRQOL had to be assessed using a validated instrument (e.g., EuroQol-5 Dimensions [39]). Only studies reporting at least one adjusted analysis (i.e., multivariable models of the exposure-outcome relationship adjusting for established confounders) were included, unless unadjusted multivariate analysis of variance (MANOVA) results were stated to be similar to adjusted multivariate analysis of covariance (MANCOVA) results. This limited uncontrolled confounding, as only non-randomized observational studies were included.

### Exclusion criteria

Studies were excluded if they were interventional, case–control studies, reviews, reports, commentaries, letters to the editor, or published in books. Studies assessing total physical activity were excluded due to inconsistent classification and lower modifiability compared to recreational activity [40–43]. Studies partially combining exposures (e.g., combining recreational and occupational activity) were excluded.

### Screening

Sources from database searches were first imported into EndNote 21 (Clarivate) reference management software, and duplicates were removed. Then, studies were imported into Covidence screening software (Veritas Health Innovation, Melbourne, Australia) for screening [44]. Three co-authors (ET, OW, KW) independently screened titles, abstracts, and full texts for eligibility. Discrepancies were resolved through discussion.

### Data charting

Data were independently extracted into Microsoft Excel by two authors (ET, OW). Extracted data included the studies’ first author, year of publication, location (i.e., country), data source, design, number of participants, patient characteristics, physical activity measurement methods, proportion of sample meeting physical activity guidelines, HRQOL measurement methods, main findings, limitations, funding source, and conflicts of interest.

### Quality assessment of individual studies

The methodological quality of each study was assessed independently by two authors (ET, OW) using the Mixed Methods Appraisal Tool (MMAT) [45], which evaluates study design, sample representativeness, measurement appropriateness, data completeness (threshold set at 80%), and potential confounding. Disagreements were resolved through discussion.

### Risk of bias assessment

The risk of bias in the included studies was assessed independently by two authors (ET, OW) using the Risk of Bias in Non-randomized Studies of Exposures (ROBINS-E) tool [46]. This tool evaluates confounding, exposure and outcome measurement, participant selection, missing data, and reporting bias. For each study, judgments of domain-specific and overall risk of bias were made according to the ROBINS-E guidelines. Disagreements were resolved through discussion.

### Certainty of evidence

The quality of the body of evidence for specific HRQOL outcomes was assessed using the Grading of Recommendations Assessment, Development and Evaluation (GRADE) approach, which evaluates how well the findings reflect the true underlying effect [47]. The approach considers factors such as risk of bias (from ROBINS-E), directness of evidence, heterogeneity, imprecision, and publication bias. Due to the greater number of studies on global and physical domains, we specifically evaluated the certainty of evidence for these two domains.

### Data synthesis and analysis

Due to the heterogeneity of physical activity measures, HRQOL instruments, and statistical methods, statistical aggregation was deemed inappropriate. Instead, a qualitative synthesis was performed. HRQOL outcomes were categorized into five domains: global, physical, emotional, social, and breast cancer-specific. Study estimates were considered clinically meaningful if the adjusted mean difference or mean difference was statistically significant and met or exceeded the established minimal important difference for the relevant HRQOL domain, which was specific to the instrument [48–54] (detailed in Supplement 3).

For studies with adjusted mean differences, results were considered favorable if increased physical activity or meeting guidelines was associated with greater HRQOL. For studies reporting adjusted means, the adjusted mean difference and 95% confidence interval (CI) were calculated. For studies reporting only unadjusted means, results were included if they were stated to be similar to adjusted analyses. Effect direction and forest plots were generated to visualize the associations.

In our secondary aim, we compared key features and findings across observational and clinical trial literature. For direct comparison, trial-based weighted mean differences were matched to adjusted mean differences from observational studies. We selected the meta-analysis by Aune et al. [18], as it provided the most recent and comprehensive analysis of clinical trial data for breast cancer survivors, including reporting by HRQOL domain (global, physical, emotional/mental).

## Results

### Study selection

**Fig. 1:**
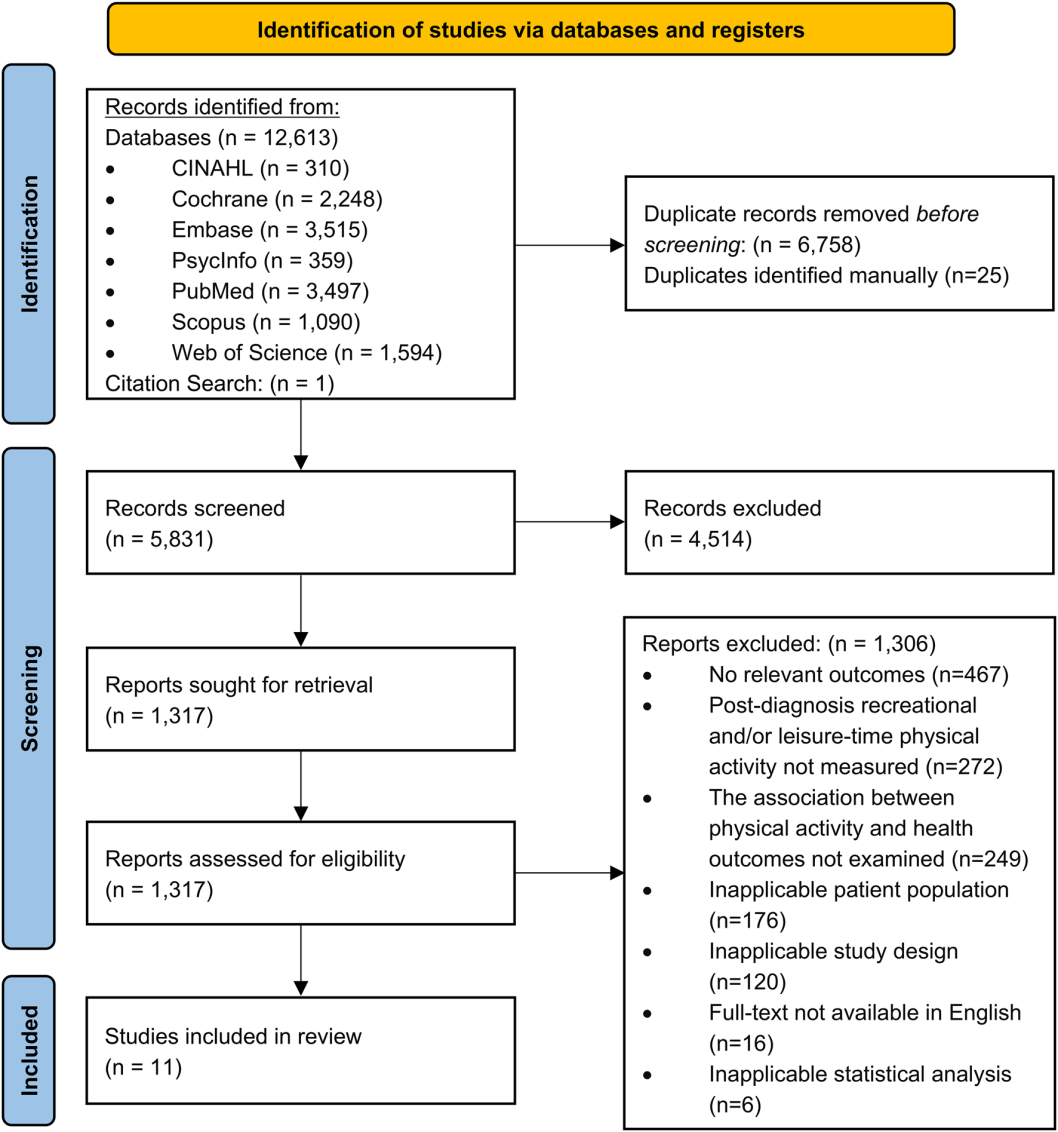
PRISMA flow diagram Note. PRISMA=Preferred Reporting Items for Systematic Reviews and Meta-Analyses

The searches identified 12,614 records, resulting in 5831 non-duplicate items. Eleven papers met the inclusion criteria and were included in this systematic review (Fig. 1). Detailed reasons for exclusion are provided in Fig. 1.

### Study characteristics

Among the 11 included studies, five were U.S.-based and six were international, representing Australia, Canada, China, and Europe. The number of breast cancer survivors included varied from 170 to 2885 (total=9254) (Table 1) [55–65]. Participants’ sociodemographic and clinical characteristics are reported in Supplements 4 and 5, respectively. Five studies were cross-sectional [61–65], and six were prospective cohort studies [55–60]. However, one prospective cohort study provided only cross-sectional analyses of the exposure-outcome association of interest [57].

**Table 1:** Study characteristics

Study	Country (N)	Outcome measure	Physical activity timing	HRQOL timing	Participants	Risk of bias^a^
**Cohort**						
Peck et al. [55]	Canada (88)	EQ5D	On-treatment	On-treatment	Receiving sequential anthracycline and trastuzumab therapy for HER2 + BC	High
					Mean ± SD = 51 ± 9 years	
					Stages I–IV	
Chen et al. [56]	China (1829)	GQLI-74	0–6 months and 18–36 months post-diagnosis	0–6 months and 18–36 months post-diagnosis	Aged 20–75 years with incident BC	Very high
					Mean ± SD = 54 ± 10 years	
Vehmanen et al. ^c^ [57]	Finland, Portugal, Israel, and Italy (311)	EORTC QLQ-C30	12-months after starting adjuvant therapy	12-months after starting adjuvant therapy	Aged 40–70 years who received adjuvant or neo-adjuvant therapy in addition to surgery	Very high
					Mean ± SD = 55 ± 8 years	
					Stages I–III	
Sagen et al. [58]	Norway (204)	EORTC QLQ-C30	6-months post-surgery	5-years post-surgery	< 75 years diagnosed with early-stage BC who underwent mastectomy or breast-conserving surgery with axillary node dissection (ALND)	Very high
					Mean ± SD = 55 ± 10 years	
					Stages I–II	
Alfano et al. [59]	US–NM, WA 545)	SF-36	Post-diagnosis	Post-diagnosis	In-situ or stages I–IIIA BC	Very high
					Mean ± SD = 58 ± 10 years	
Hart et al. [60]	US–WI (1448)	SF-36	Post-diagnosis	Post-diagnosis	Diagnosed with ductal carcinoma in situ, aged 20–74 years at diagnosis	High
					Mean ± SD = NR	
**Cross-sectional**						
Milne et al. ^b^ [61]	Australia (558)	FACT-B; FACT-G	Post-treatment	Post-treatment	> 18 years diagnosed with BC in 2002, no longer undergoing active treatment and with no secondary cancers	Very high
					Mean ± SD = 59 ± 11 years	
					Stages I–IV	
Vallance et al. ^b^ [62]	Canada (524)	FACT-G	Post- & on-treatment separately	Post-treatment	≥ 18 years living in rural and small towns and completed adjuvant therapy except hormone therapy	High
					Mean ± SD = 62 ± 11 years	
					Stages I–III	
Blanchard et al. [63]	US (2885)	SF-36	Long-term post-diagnosis	Long-term post-diagnosis	≥ 18 years diagnosed with local, regional, or distant BC in the calendar year either 2, 5, or 10 years before sampling	Very high
					Mean = 63 ± 12 years	
Dibble et al. [64]	US–CN (170)	FACT-B	Post-treatment	Post-treatment	Postmenopausal who completed treatment	Very high
					Mean = 69 ± 10 years	
					Stages I–III	
Pakiz et al. [65]	US (692)	SF-36, IOC	Post-treatment	Post-treatment	Overweight or obese aged ≥ 21 years, diagnosed within the previous 5 years, and who have completed initial therapies (excluding endocrine therapy) at entry into a weight loss trial, prior to receiving the trial intervention	Very high
					Mean = 56 ± 9 years Stages I–III	

*HRQOL* Health-related quality of life

^a^Per Risk Of Bias In Non-randomized Studies—of Exposure (ROBINS-E) assessment detailed in above table

^b^Retrospective cross-sectional studies

^c^Prospective cohort study reporting cross-sectional analysis

The studies used seven different HRQOL questionnaires, including both generic and disease-specific instruments. Generic instruments—36-Item Short Form Survey (SF-36), European Organization for Research and Treatment of Cancer Quality of Life Questionnaire Core 30 (EORTC QLQ-C30), the three-level EuroQol five-dimensional questionnaire (EQ-5D-3L), Functional Assessment of Cancer Therapy—General (FACT-G), the General Quality of Life Inventory-74 (GQLI-74), and the Impact of Cancer Scale (IOC)—were exclusively used in nine studies [39, 66–68]. A disease-specific instrument, the Functional Assessment of Cancer Therapy – Breast (FACT-B), was exclusively used in one study [64], while another study reported on both FACT-B and FACT-G [6]. FACT-B is an extension of the generic FACT-G, incorporating an additional breast cancer-specific subscale. A summary of the most commonly used instruments is provided in Supplement 3.

Supplement 6 reports the instruments used in the included studies to measure recreational physical activity and the proportion of survivors meeting activity guidelines. The Godin Leisure Time Exercise Questionnaire, which has been validated in classifying the activity level of breast cancer survivors [69], was the most frequently used physical activity measure (n = 4) [70]. Other measures used in single studies included the Modifiable Activity Questionnaire [71], Shanghai Women’s Health Study Physical Activity Questionnaire [72], National Health and Nutrition Examination Survey items [73], and Nurses’ Health Study II items [74]. Two studies used unnamed measures for recreational activity [57, 58].

All studies included in this review reported aerobic activity. Among studies reporting the proportion of participants meeting physical activity guidelines, 31–66% of breast cancer survivors met aerobic activity guidelines (≥ 150 min/week of moderate-intensity aerobic activity) [55–57, 61–64]. Dibble et al. [64] (the singular study reporting muscle-strengthening in addition to aerobic activity) found that 36.5% of survivors met muscle-strengthening guidelines (≥ 2 days/week).

### Methodological quality

**Fig. 2:**
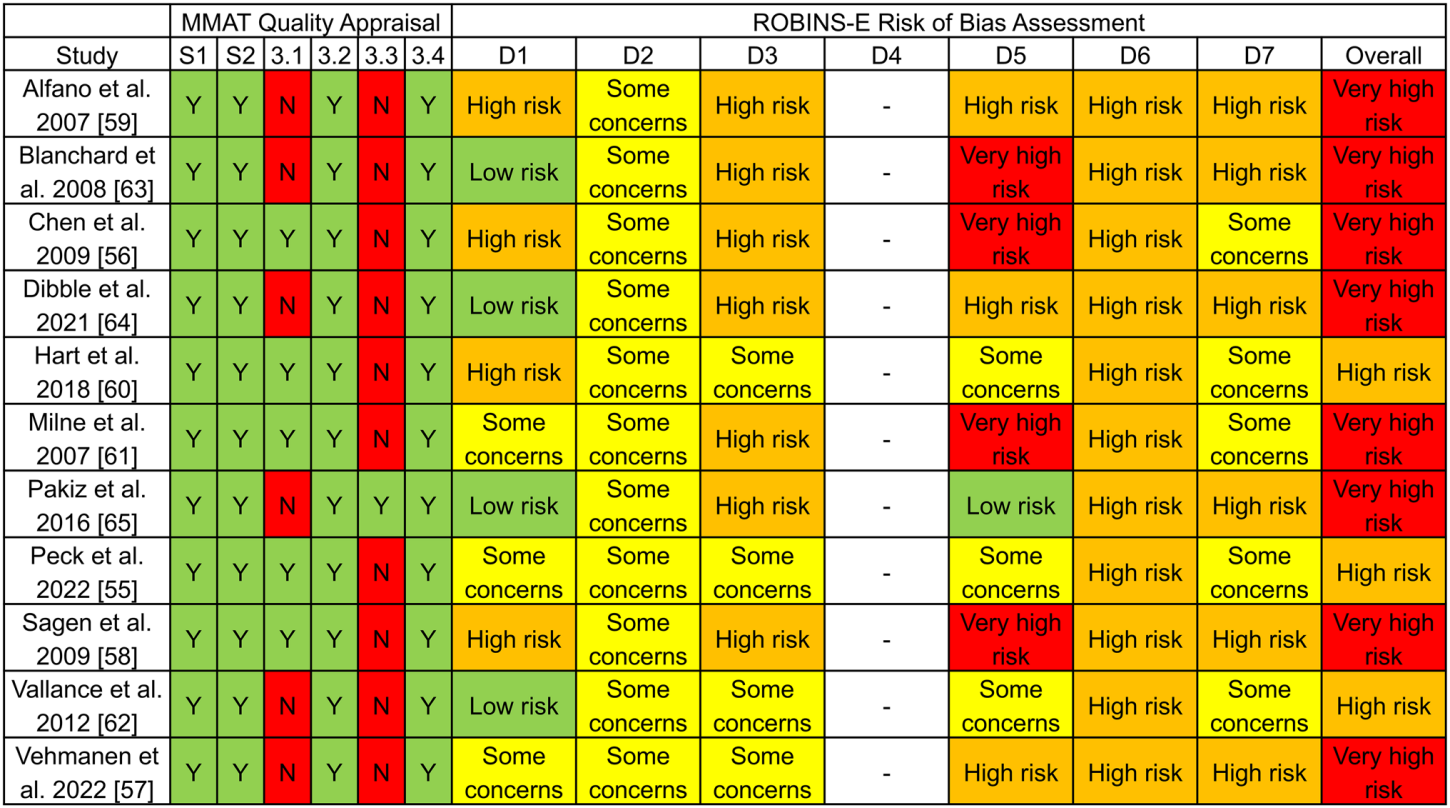
Quality appraisal and risk of bias assessment Notes. S1: Are research question(s) clear?; S2: Is data collection appropriate to answer research question(s)?; 3.1: Are participants representative of target population?; 3.2: Are measurements appropriate?; 3.3: Is outcome data complete?; 3.4 Are confounders accounted for in design and/or analysis?; D1: Confounding; D2: Exposure Measurement; D3: Participant Selection into Study/Analysis; D4: Post-Exposure Interventions; D5: Missing Data; D6: Outcome Measurement; D7: Reporting Notes: MMAT = Mixed Methods Appraisal Tool; ROBINS-E = Risk Of Bias In Non-randomized Studies - of Exposure; Y = Yes; N = No

The evaluation of methodological quality and risk of bias for the 11 studies is shown in Fig. 2. All studies had a clear research question, collected appropriate data, used suitable measurements, and accounted for confounders in the design or analysis. Methodological shortcomings mainly related to the representativeness of the study sample to the target population for six studies, as well as incomplete outcome data for ten studies.

### Risk of bias

The risk of bias evaluation is shown in Fig. 2. High to very high risk of bias was prevalent in participant selection and analysis, missing data, outcome measurement, and reporting bias. For overall risk of bias, eight studies were rated as “very high risk” [56–59, 61, 63–65], and three studies as “high risk” [55, 60, 62]. No studies were classified as “some concerns” or “low risk.” Additional details on the risk of bias evaluation are provided in Supplement 7.

### Associations between physical activity and HRQOL

**Fig. 3:**
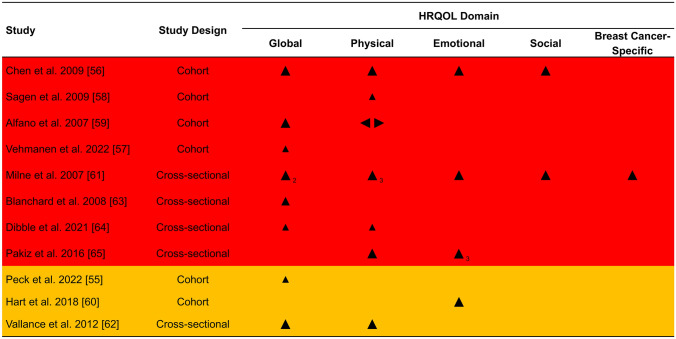
Effect direction plot summarizing direction of outcome domains of included studies Notes. Subscript indicates number of outcomes within the HRQOL domain reported in the respective study Effect direction: upward arrow ▲= positive health impact, downward arrow ▼= negative health impact, sideways arrow ◄►= no change/mixed effects/conflicting findings Sample size: Final sample size (individuals) in exposure group Large arrow ▲ >300; medium arrow ▲ 50-300; small arrow ▲ <50 Study quality: denoted by row color: yellow = some concerns; orange = high risk of bias; red = very high risk of bias

**Fig. 4:**
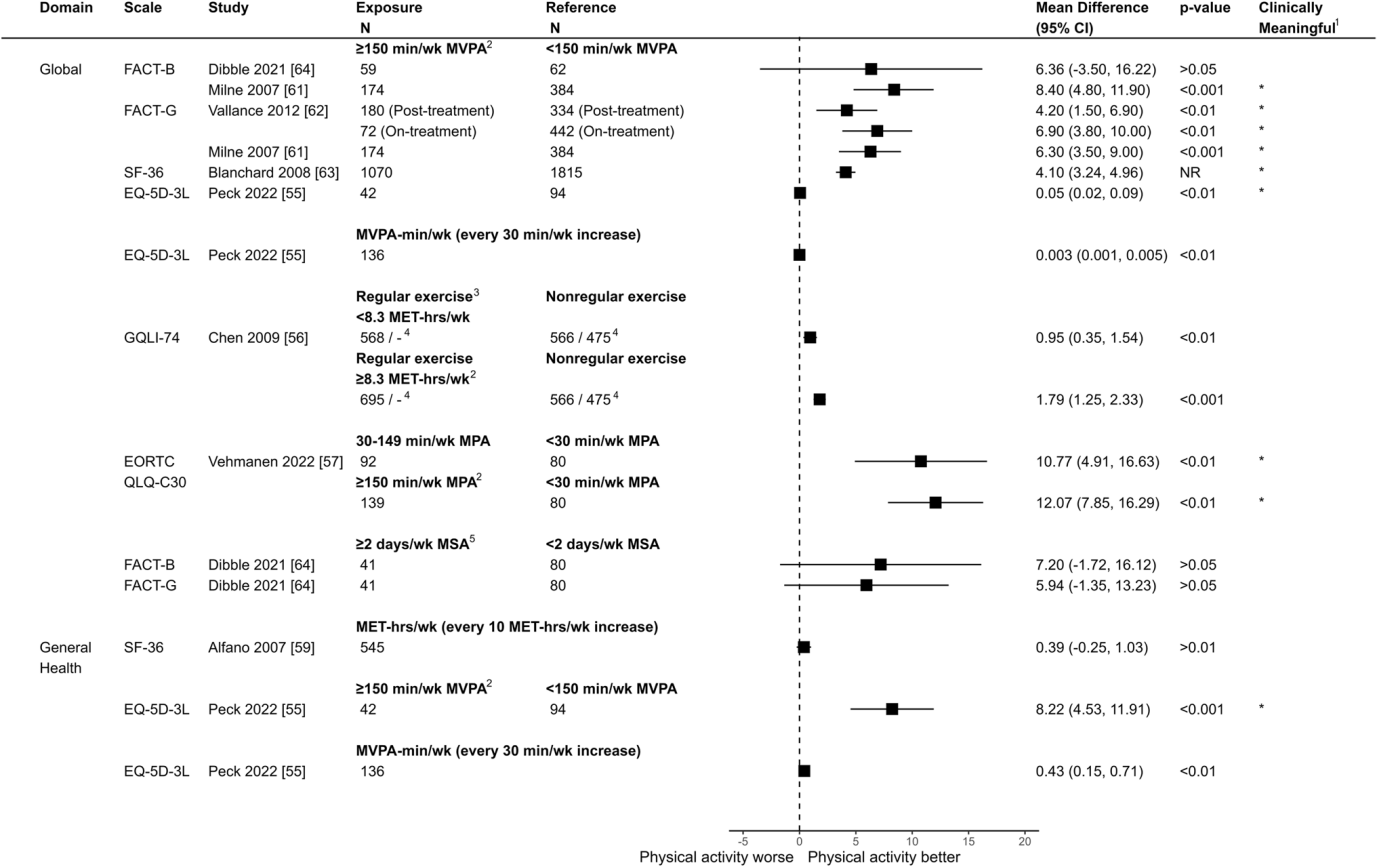
Comparison of adjusted mean differences of global/total HRQOL between breast cancer survivors by physical activity level (exposure vs. reference) Notes. ^1^Clinically meaningful was defined as a mean difference exceeding the minimum important difference threshold (see Supplement 3) and being statistically significant. ^2^Corresponds to meeting aerobic activity guidelines. ^3^Regular exercisers defined as individuals reporting exercising at least twice per week, while non-regular exercisers were those reporting exercising less than twice per week. ^4^Results are derived from a mixed model. Sample sizes are reported for the 6-month and 36-month follow-up periods. ^5^Corresponds to meeting muscle-strengthening activity guidelines. CI = Confidence Interval; FACT-B = Functional Assessment of Cancer Therapy - Breast; FACT-G = Functional Assessment of Cancer Therapy - General; SF-36 = 36-Item Short Form Health Survey; GQLI-74 = Generic Quality of Life Inventory - 74; EORTC QLQ-C30 = European Organization for Research and Treatment of Cancer Quality of Life Questionnaire - Core 30; MVPA = Moderate-to-Vigorous Physical Activity, MET-hrs/wk = Metabolic Equivalent of Task Hours per Week; MPA = Moderate Physical Activity; MSA = Muscle-strengthening Activity

The overall direction of association was positive in 21/22 (95%) of domains reported (Fig. 3). Only one study reported a small negative association for role functioning [59]. For all outcomes across HRQOL domains, sample sizes for each group (when reported) are presented in Figs. 4 (global HRQOL) and 5 (physical HRQOL) and Supplements 8-10 (social, emotional, and breast cancer-specific HRQOL). Adjusted mean difference (95% CI) are reported in forest plots, with adjusted mean difference > 0 indicating a positive association between physical activity and the outcome. Summaries of domain-specific HRQOL associations with recreational physical activity are presented in Supplements 11-15.

### Global HRQOL

Seven studies reported on global HRQOL [55–57, 61–64], while two reported on general health perception [55, 59]. All studies found a positive association between physical activity, whether aerobic or muscle-strengthening, and global HRQOL, ranging from small/trivial to clinically meaningful adjusted mean differences. Seven comparisons across four studies [55, 61–63] found significant positive associations between meeting aerobic physical activity guidelines and global HRQOL (Fig. 4 & Supplement 11). For instance, Milne et al. [61] found that breast cancer survivors meeting aerobic activity guidelines reported, on average, a global HRQOL score 8.40 (95% CI: 4.80, 11.90) points higher on FACT-B compared to those not meeting aerobic activity guidelines. Similarly, Vallance et al. [62], Blanchard et al. [63], and Peck et al. [55] reported clinically meaningful positive associations between meeting aerobic activity guidelines and global HRQOL, as measured by FACT-G, SF-36, and EQ-5D-3L, respectively (Fig. 4).

**Fig. 5:**
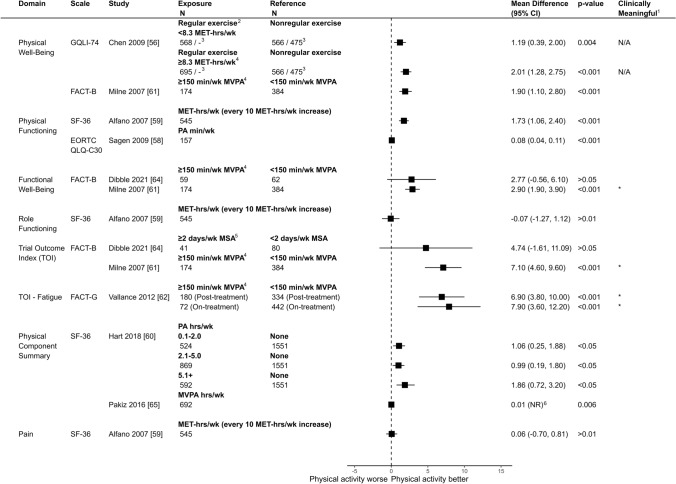
Comparison of adjusted mean differences of physical well-being and function domains of HRQOL between breast cancer survivors by physical activity level (exposure vs. reference) Notes. ^1^Clinically meaningful was defined as a mean difference exceeding the minimum important difference threshold (see Supplement 3) and being statistically significant. ^2^Regular exercisers defined as individuals reporting exercising at least twice per week, while non-regular exercisers were those reporting exercising less than twice per week. ^3^Results are derived from a mixed model. Sample sizes are reported for the 6-month and 36-month follow-up periods. ^4^Corresponds to meeting aerobic activity guidelines. ^5^Corresponds to meeting muscle-strengthening activity guidelines. ^6^Results derived from a linear regression of a log-transformed outcome CI = Confidence Interval; FACT-B = Functional Assessment of Cancer Therapy - Breast; FACT-G = Functional Assessment of Cancer Therapy - General; SF-36 = 36-Item Short Form Health Survey; GQLI-74 = Generic Quality of Life Inventory - 74; EORTC QLQ-C30 = European Organization for Research and Treatment of Cancer Quality of Life Questionnaire - Core 30; MVPA = Moderate-to-Vigorous Physical Activity; MET-hrs/wk = Metabolic Equivalent of Task Hours per Week; MPA = Moderate Physical Activity; MSA = Muscle-strengthening Activity; PA = Physical Activity

Dibble et al. [64] found higher global HRQOL for those meeting muscle-strengthening guidelines, although the 95% CI included the null value (Adjusted Mean Difference: 7.20, 95% CI: − 1.72, 16.12 for FACT-B, and 5.94, 95% CI: − 1.35, 13.23 for FACT-G). For general health perception, Peck et al. [55] reported a clinically meaningful improvement exceeding the minimal important difference (Adjusted Mean Difference: 8.22; 95% CI: 4.53–11.91) associated with aerobic activity, whereas Alfano et al. [59] observed a minimal, non-significant increase that did not meet this threshold (Adjusted Mean Difference: 0.39; 95% CI: − 0.25–1.03).

### Physical function and well-being

Eight studies reported on physical HRQOL subdomains [56, 58–62, 64, 65] (Fig. 5 & Supplement 12). Point estimates associated with aerobic or muscle-strengthening activity ranged from small/trivial to clinically meaningful improvements [56, 58–60, 62, 64, 65], with the exception of Milne et al. [61], who reported a small negative association between meeting aerobic activity guidelines and role functioning (Adjusted Mean Difference: − 0.07, 95% CI: -1.27, 1.12). Four comparisons across two studies demonstrated clinically meaningful positive associations between aerobic activity and physical subdomains of HRQOL. For example, Milne et al. [61] observed improved functional well-being and trial outcome index (TOI) scores associated with meeting aerobic activity guidelines (Adjusted Mean Difference: 2.90, 95% CI: 1.90, 3.90 and Adjusted Mean Difference: 7.10, 95% CI: 4.60, 9.60). Similarly, Vallance et al. [62] found improved TOI – Fatigue scores both on- and post-treatment (Adjusted Mean Difference: 7.90, 95% CI: 3.60, 12.20 and Adjusted Mean Difference: 6.90, 95% CI: 3.80, 10.00). For muscle-strengthening activity, Dibble et al. [64] found higher TOI scores associated with meeting guidelines, though the 95% CI included the null (Adjusted Mean Difference: 4.74, 95% CI: − 1.61, 11.09).

### Emotional function and well-being

Four studies reported on emotional subdomains of HRQOL, all finding positive point estimates for the association between aerobic activity and emotional, psychological, and mental health HRQOL outcomes (Supplements 8 & 13) [56, 60, 61, 65]. However, for these results, the estimates either did not reach the threshold for a minimal important difference, or the threshold values for the respective HRQOL scales were unavailable.

### Social well-being

Two studies reported on social well-being, and both found positive associations between aerobic activity and social well-being (Supplements 9 & 14) [56, 61]. Chen et al. [56] found that regular exercisers (exercising at least twice per week), whether or not they met aerobic exercise guidelines, had significantly improved social well-being at 6 and 36 months compared to non-regular exercisers. The adjusted mean differences were 0.99 (95% CI: 0.25, 1.74) for regular exercisers not meeting the guidelines, and 2.21 (95% CI: 1.54, 2.88) for those meeting the guidelines. Milne et al. [61] found a modest positive association between meeting aerobic activity guidelines and HRQOL in breast cancer survivors, although this did not reach the minimal important difference threshold (Adjusted Mean Difference: 0.70, 95% CI: − 0.20, 1.70).

### Breast cancer subscale

Only one study reported HRQOL on the Breast Cancer Subscale (FACT-B), where those meeting aerobic activity guidelines reported higher scores compared to those not meeting guidelines, and this difference was clinically meaningful (Adjusted Mean Difference: 2.20, 95% CI: 1.20, 3.40) (Supplements 10 & 15) [61].

### Grading of evidence

Evidence was graded according to GRADE criteria (Supplement 16). While multiple observational studies provided evidence, the strength of associations was generally small to trivial. The evidence suggests that meeting aerobic activity guidelines may be associated with improved global HRQOL and reduced fatigue (TOI-F). However, observational data are insufficient to determine whether physical activity, when unprompted by intervention, is associated with improved physical functioning, physical well-being, and other HRQOL domains.

### Comparison to meta-analysis of randomized controlled trials

Table 2 outlines observational and trial study designs and compares the observational evidence from our review with randomized controlled trial data from a meta-analysis by Aune et al. [18]. In terms of design, observational studies assess real-world activity, while trials typically administer an intervention and compare to a control arm. For instance, while a trial may deliver a 12 week supervised program, observational data may reflect free-living activity. Both study types rely on self-reported HRQOL, which may introduce bias [75]. Trials reduce confounding through randomization but may have limited external validity due to homogeneous populations, whereas observational studies, while more prone to uncontrolled confounding, offer improved external validity with more diverse populations.

**Table 2:** Comparison of randomized controlled trial and observational study designs and findings on the association between physical activity and HRQOL among breast cancer survivors

	Randomized Controlled Trial	Observational
Findings	Weighted mean difference (95% CI)	Adjusted mean difference (95% CI)
	**FACT-B**	**FACT-B**
	Aerobic: 3.85 (1.14, 6.57)	Aerobic^b^: 6.36–8.40 (− 3.50, 16.22)
	Muscle-strengthening: NR^a^	Muscle-strengthening^b^: 7.20 (− 1.72, 16.12)
	**FACT-G**	**FACT-G**
	Aerobic: 2.88 (0.86, 4.90)	Aerobic^b^: 4.20–6.90 (1.50, 10.00)
	Muscle-strengthening: 4.35 (− 1.47, 10.17)	Muscle-strengthening^b^: 5.94 (− 1.35, 13.23)
	**EORTC QLQ-C30**	**EORTC QLQ-C30**
	Aerobic: 8.79 (− 1.56, 19.14)	Aerobic^c^: 10.77–12.07 (4.91, 16.63)
	Muscle-strengthening: 5.21 (1.47, 8.96)	Muscle-strengthening: NR^a^
Study design	Trial participants randomized to an intervention or control (e.g., usual care) arm	Cross-sectional or longitudinal cohort studies
		Study participants are not randomized to physical activity levels
Study population	Narrow, homogenous (more likely to be highly motivated, healthy participants which may lead to healthy adherer bias)	More representative, heterogenous
Causal inference	Observed associations are more likely to be causal relationships	Less likely to be causal due to unmeasured confounders (e.g., general health) or lack of temporality in cross sectional studies
Exposure	Designed physical activity interventions varying in frequency and intensity (i.e., dose) and type (e.g., aerobic, resistance, stretching, balance), either alone or in combination	Measured physical activity varying in terms of frequency (e.g., days/week), intensity (e.g., moderate), duration (e.g., hours), and type (e.g., aerobic and/or resistance activity levels)
		Frequently categorized into meeting vs not meeting physical activity guidelines
Physical activity behavior	Often provide supervision and/or scheduled sessions	Capture a broader range of physical activity, including more self-directed or sporadic activity, with some participants potentially engaging in supervised training programs resembling those in trials
Outcome	Patient-reported HRQOL using disease-specific and generic instruments	
Analysis	Direct comparison of outcomes across trial arms	Requires multivariable techniques to account for confounding
Internal validity	High	Low to moderate
External validity/generalizability	Low to moderate	Moderate to high
Strengths	Randomization may minimize confounding by unmeasured and measured factors	Can study large populations
		Statistical methods (e.g., propensity score matching) may address confounding due to measured factors
		Less costly
Limitations	Limited generalizability due to recruitment and selection bias	Unmeasured confounding (e.g., baseline HRQOL)
	More costly	Cross-sectional design (unable to establish temporality, potential reverse causation)

^a^No estimate reported for muscle-strengthening activity

^b^Meeting guidelines

^c^Range of estimates for 30–149 min/week and ≥ 150 minutes/week of moderate physical activity compared to < 30 min/week of physical activity

Notes. NR = Not Reported

We compared instrument-specific weighted mean differences (95% CI) for global HRQOL from physical activity interventions pooled in Aune et al. [18] with findings from observational studies (Table 2). Point estimates for aerobic-only interventions in Aune et al. [18] were lower than those observed for meeting aerobic activity guidelines in observational studies across FACT-B, FACT-G, and EORTC QLQ-C30, although confidence intervals overlapped. FACT-G was the only scale that included muscle-strengthening data from both trials and observational studies, and there was considerable overlap in estimates for meeting muscle-strengthening guidelines (observational) and muscle-strengthening-only interventions (trials). Comparisons for general health perception, physical, and mental HRQOL showed positive estimates in both trial and observational studies, with varying magnitudes and precision (Supplements 17–20). For these domains, there is a general consistency across trials and observational studies given the considerable overlap in confidence intervals and the consistent positive direction of the point estimates. A comparison for social or breast cancer-specific HRQOL was not possible, as no meta-analysis for these domains was identified.

## Discussion

Clinical trials have shown that physical activity interventions improve HRQOL. This review aimed to assess the association between recreational physical activity, unprompted by trial intervention, and HRQOL in a heterogeneous sample. Our results, summarizing data from 9254 women across 11 observational studies, suggest recreational aerobic activity may be positively associated with global, physical, and breast cancer-specific HRQOL, aligning with clinical trial meta-analyses [13, 18, 19]. However, key gaps remain, including limited data on emotional and social HRQOL, muscle-strengthening activity, and optimal activity dosage for individual patients.

To our knowledge, this is one of the first studies to compare the relationship between physical activity and HRQOL across observational studies and trials. The combined evidence from both experimental and observational studies supports the inherent benefits of aerobic activity in improving well-being, likely through a range of mechanisms. While both structured exercise interventions in trials and daily life activity likely share similar physiological effects, the contexts in which they occur can differ. Trials often provide supervision and/or scheduled sessions, whereas observational studies capture a broader range of physical activity behaviors, including more self-directed or sporadic activity, with some participants potentially engaging in supervised training programs resembling those in trials. Regardless, individuals engaging in aerobic activity, whether structured or informal, experience similar physiological benefits associated with improved HRQOL [76]. There are several physiological hypotheses surrounding how physical activity may improve mental and physical health, including the endorphin hypothesis and reduced Hypothalamic–Pituitary–Adrenal (HPA) axis response to stress [77]. For instance, activity increases interleukin-6, boosting anti-inflammatory cytokine secretion and dampening the HPA axis response to stress, potentially alleviating mental stress and preventing conditions associated with systemic inflammation (e.g., obesity, cardiovascular disease) [78, 79].

This study has several strengths, including being the first to synthesize observational data on HRQOL among breast cancer survivors and compare these findings with clinical trials. We also synthesized analyses on the association between meeting aerobic and muscle-strengthening guidelines and HRQOL. However, our study has limitations. First, the evidence base was limited, with only 11 studies. While the observational studies had larger sample sizes than typical trials (range 88–2,885 vs. 20–573 in Aune et al.’s meta-analysis), many were underpowered to detect small effect sizes and did not meet GRADE guidelines’ optimal information size [80]. There were also limitations pertaining to physical activity measurement and analysis. For instance, we lacked objectively measured physical activity (e.g., accelerometer data), as studies with such data focused on total rather than recreational activity and were excluded. Further, although most studies (9/11) used validated physical activity measures, these tools have limitations. For instance, the Godin Leisure-Time Exercise Questionnaire captures frequency of 15 min sessions but does not quantify precise activity levels. The two studies lacking validated physical activity measures were still included because they focused on recreational activity and used validated HRQOL measures. Lastly, most studies reported only a binary classification of whether participants met physical activity guidelines, limiting the insight into how varying doses of activity are associated with HRQOL.

In addition, there were issues related to the observational studies’ methodology. First, six studies employed cross-sectional analysis, so temporality could not be established. Second, the extended latency between follow-ups in cohort studies, such as those measuring HRQOL years after physical activity data collection, made it difficult to infer causality. Third, there may have been unmeasured confounding. For instance, most studies did not control for baseline HRQOL, limiting our ability to determine whether changes in HRQOL are attributable to physical activity. Thus, the higher point estimates observed in observational studies, compared to those in Aune et al.’s meta-analysis [18], may reflect the effects of lack of adjustment for unequally distributed baseline characteristics. Future studies should prioritize adjusting for baseline HRQOL and other characteristics (e.g., hormone sensitivity). Fourth, many studies did not report sensitivity analyses or address missing data, which could have mitigated bias from nonresponse, selection, and loss to follow-up. Fifth, Hart et al.’s study, which focused exclusively on women with ductal carcinoma in situ, a non-invasive form of breast cancer, may be limited in comparability to studies involving invasive breast cancer. Sixth, inconsistent reporting of HRQOL domains limited cross-study comparability. Some studies selectively reported significant results or omitted domains, leading to weaker evidence for emotional and social HRQOL compared to global and physical domains. Transparent reporting, such as including non-significant findings in supplementary materials, would strengthen the evidence base. Finally, we could not assess the clinical significance of findings from two studies due to the lack of published minimal important difference estimates for the HRQOL scales used. Future studies should use validated scales with established minimal important differences or incorporate clinical anchors, such as performance status or patient-reported health changes. Alternatively, reporting effect size (e.g., Cohen’s d, eta-squared, r-squared) provides a standardized measure of the magnitude of the effect (small, medium, large), as opposed to presenting only point estimates from regression analyses, which are specific to the outcome measurement scale. This approach enhances clinical translation, as the focus should not be limited to the statistical significance of an association, but also on whether the observed differences are meaningful to patients.

Our findings highlight key considerations for future observational research. The temporal relationship between physical activity and HRQOL could be assessed using longitudinal, prospective cohort designs with repeated measures of physical activity and HRQOL. Additionally, recruiting a large, representative sample accounting for sociodemographic and clinical factors may help improve generalizability of study findings. To address the current gap in evidence, we encourage future studies to assess muscle-strengthening activities in addition to aerobic activity. While we excluded studies using objective measures (e.g., accelerometers) due to inconsistent domain measurement (recreational vs. total vs. occupational), increased use of objective activity measures with consistent domain classifications would reduce self-report bias. For HRQOL, using validated, uniform HRQOL instruments would improve cross-study comparability. Collecting data on potential confounders such as baseline HRQOL, age at diagnosis, disease stage, treatment history, and socioeconomic factors would strengthen analytic rigor. When analyzing the exposure-outcome relationship, examining varying doses of physical activity, along with plotting activity levels against HRQOL, would offer more insight into the nature of the relationship between physical activity dosage and HRQOL. Robust statistical methods such as multiple imputation and sensitivity analyses should be used to handle missing data and minimize bias from nonresponse. Finally, statistical analyses and result reporting should adhere to guidelines such as Strengthening the Reporting of Observational Studies in Epidemiology (STROBE) [81] and include transparent presentation of both significant and nonsignificant results.

## Conclusions

The observational evidence suggests that recreational physical activity may be associated with clinically meaningful improvements in global and physical HRQOL. Among survivors meeting aerobic activity guidelines, improvements in global HRQOL of 10.77–12.07 points were observed on the EORTC QLQ-C30 instrument [57]. These findings are consistent with trial-based meta-analyses. For clinicians and policymakers, these findings suggest that encouraging survivors to meet at least aerobic activity guidelines may lead to clinically meaningful improvements in HRQOL. Given their larger scope, observational studies have significant potential to inform the optimal prescription of physical activity for breast cancer survivors. However, this potential has yet to be fully realized due to limitations in study design and execution. Future studies should consider including sensitivity analyses for missing data, controlling for baseline confounding variables (including baseline HRQOL in cohort studies), exploring the shape of the dose–response curve for both aerobic and muscle-strengthening activity, and providing summaries of key HRQOL domains (e.g., global, physical, and emotional subscales).

## Supplementary Information

Below is the link to the electronic supplementary material.

Supplementary file1 (Docx 955 KB)

## Data Availability

Data sharing is not applicable to this article as no datasets were analyzed or generated during the current study. All the studies summarized in this review are clearly identified within the article.

## Declarations

### Disclaimer

The contents and views in this manuscript are those of the authors and should not be construed to represent the views of the National Institutes of Health. Opinions and comments expressed in this paper belong to the authors and do not necessarily reflect those of the U.S. Government, Department of Health and Human Services, National Institutes of Health, or the National Institute on Minority Health and Health Disparities. The study funders had no role in the design of the study, the collection, analysis, or interpretation of the data, the writing of the manuscript, or the decision to submit the manuscript for publication.
