# Supplementary material for: Recreational physical activity and health-related quality of life among breast cancer survivors: a systematic review
Source: Qual Life Res. 2025 Jul 2;34(9):2513–29. doi: 10.1007/s11136-025-03992-1 (PMC12323690; doi:10.1007/s11136-025-03992-1)
Supplement: Supplementary file 1 — Supplementary file1 (Docx 955 KB) [file 11136_2025_3992_MOESM1_ESM.docx]

**Supplement 1.** Search strategies

Database: PubMed/MEDLINE
Platform: National Library of Medicine
Date Searched: 10/04/24

Date Limits: 1/1/2003 – 10/04/24

|  | Concept: | Search Strategy: |
| --- | --- | --- |
| #1 | Breast Cancer | "Breast Neoplasms"[Mesh] OR (("Breast"[Mesh] OR "breast*"[Title/Abstract]) AND ("cancer*"[Title/Abstract] OR "neoplas*"[Title/Abstract] OR "carcinoma*"[Title/Abstract] OR "tumor*"[Title/Abstract] OR "tumour*"[Title/Abstract] OR "malignan*"[Title/Abstract] OR "sarcoma*"[Title/Abstract] OR "lymphoma*"[Title/Abstract])) |
| #2 | Physical Activity | "Exercise"[Mesh] OR "Exercise Movement Techniques"[Mesh] OR "Exercise Therapy"[Mesh] OR "exercise*"[Title/Abstract] OR "Physical Fitness"[Mesh] OR "physical fitness"[Title/Abstract] OR "physical activit*"[Title/Abstract] OR "physical performanc*"[Title/Abstract] OR "Physical Exertion"[Mesh] OR "physical exertion"[Title/Abstract] OR "Physical Endurance"[Mesh] OR "physical endurance"[Title/Abstract:~4] OR "endurance training"[Title/Abstract] OR "physical intensit*"[Title/Abstract] OR "Recreation"[Mesh:NoExp] OR "recreational activit*"[Title/Abstract] OR "Sports"[Mesh] OR "sport*"[Title/Abstract] OR "stretching"[Title/Abstract] OR "Yoga"[Mesh] OR "yoga"[Title/Abstract] OR "Tai Ji"[Mesh] OR "Tai Ji"[Title/Abstract] OR "Tai Chi"[Title/Abstract] OR "muscle strength*"[Title/Abstract] OR "muscle strengthening"[Title/Abstract:~4] OR "resistance training"[Title/Abstract] OR "strength training"[Title/Abstract:~4] OR "muscle strength*"[Title/Abstract] OR "weight training"[Title/Abstract] OR "weightlifting"[Title/Abstract] OR "weight lifting"[Title/Abstract] OR "Walking"[Mesh] OR "walk*"[Title/Abstract] OR "gait"[Title/Abstract] OR "jog"[Title/Abstract] OR "jogging"[Title/Abstract] OR "running"[Title/Abstract] OR "swimming"[Title/Abstract] OR "aerobic*"[Title/Abstract] OR "exertion*"[Title/Abstract] OR "isometric*"[Title/Abstract] OR "energy expenditure*"[Title/Abstract] OR "energy balanc*"[Title/Abstract] OR "energy intake*"[Title/Abstract] OR "circuit*"[Title/Abstract] OR "high intensity interval*"[Title/Abstract] OR "high intensity intermittent*"[Title/Abstract] OR "HIIT"[Title/Abstract] OR "sprint interval*"[Title/Abstract] OR "stair climbing"[Title/Abstract:~3] OR "calisthenic*"[Title/Abstract] OR "cycling*"[Title/Abstract] OR "biking*"[Title/Abstract] OR "Sedentary Behavior"[Mesh] OR "sedentary"[Title/Abstract] OR "physical inactivit*"[Title/Abstract] |
| #3 | Mortality & Recurrence | "Neoplasm Recurrence, Local"[Mesh] OR "Recurrence"[Mesh] OR "recurren*"[Title/Abstract] OR "reoccurren*"[Title/Abstract] OR "relaps*"[Title/Abstract] OR "Neoplasms, Second Primary"[Mesh] OR "secondary events"[Title/Abstract] OR "second cancer*"[Title/Abstract] OR "second primary cancer*"[Title/Abstract] OR "secondary cancer*"[Title/Abstract] OR "secondary primary cancer*"[Title/Abstract] OR "Disease Progression"[Mesh] OR "progression*"[Title/Abstract] OR "Disease-Free Survival"[Mesh] OR "cancer survivor*"[Title/Abstract] OR "cancer survival"[Title/Abstract:~3] OR "cause death"[Title/Abstract:~3] OR "all cause"[Title/Abstract] OR "fatal*"[Title/Abstract] OR "Mortality"[Mesh] OR "mortality" [Subheading] OR "mortalit*"[Title/Abstract] OR "Quality of Life"[Mesh] OR "quality of life"[Title/Abstract] OR "life quality"[Title/Abstract] OR "HRQOL"[Title/Abstract] |
| #4 | Limits & Filters | ((#1 AND #2 AND #3) NOT ("Animals"[Mesh] NOT ("Animals"[Mesh] AND "Humans"[Mesh]))) NOT (editorial[Publication Type] OR comment[Publication Type] OR "commentary*"[Title/Abstract] OR news[Publication Type] OR letter[Publication Type] OR retracted publication[Publication Type] OR retraction of publication[Publication Type] OR "retraction of publication*"[Title/Abstract] OR "retraction notice"[Title] OR "retracted publication"[Title] OR "Congress"[Publication Type] OR "Consensus Development Conference"[Publication Type] OR "conference abstract*"[Title/Abstract] OR "conference proceeding*"[Title/Abstract] OR "conference paper*"[Title/Abstract] OR "conference review*"[Title/Abstract] OR "symposium*"[Title/Abstract] OR "Case Reports" [Publication Type] OR "case report*"[Title/Abstract] OR "Review" [Publication Type] OR "review"[Title] OR "Systematic Review" [Publication Type] OR "Systematic Reviews as Topic"[Mesh] OR "systematic review"[Title/Abstract] OR "Meta-Analysis" [Publication Type] OR "Meta-Analysis as Topic"[Mesh] OR "meta-analysis "[Title/Abstract] OR "meta-analyses "[Title/Abstract] OR "protocol"[Title] OR "protocols"[Title] OR "Practice Guideline"[Publication Type] OR "guideline*"[Title]) Filters: English, from 2003/1/1 - 2023/9/18 |

Database: Cochrane CENTRAL
Platform: Wiley & Sones
Date Searched: 10/04/24

Date Limits: 1/1/2003 – 10/04/24

|  | Concept: | Search Strategy: |
| --- | --- | --- |
| #1 | Breast Cancer | ([mh "Breast Neoplasms"] OR (([mh "Breast"] OR breast*) NEAR/4 (cancer* OR neoplas* OR carcinoma* OR tumor* OR tumour* OR malignan* OR sarcoma* OR lymphoma*))):ti,ab,kw |
| #2 | Physical Activity | ([mh "Exercise"] OR [mh "Exercise Movement Techniques"] OR [mh "Exercise Therapy"] OR exercise* OR [mh "Physical Fitness"] OR "physical fitness" OR (physical NEXT activit*) OR (physical NEXT performanc*) OR [mh "Physical Exertion"] OR "physical exertion" OR [mh "Physical Endurance"] OR (physical NEAR/4 endurance) OR "endurance training" OR (physical NEXT intensit*) OR [mh ^"Recreation"] OR (recreational NEXT activit*) OR [mh "Sports"] OR sport* OR stretching OR [mh "Yoga"] OR yoga OR [mh "Tai Ji"] OR "Tai Ji" OR "Tai Chi" OR (muscle NEXT strength*) OR (muscle NEAR/4 strengthening) OR "resistance training" OR (strength NEAR/4 training) OR (muscle NEXT strength*) OR "weight training" OR weightlifting OR "weight lifting" OR [mh "Walking"] OR walk* OR gait OR jog OR jogging OR running OR swimming OR aerobic* OR exertion* OR isometric* OR (energy NEXT expenditure*) OR (energy NEXT balanc*) OR (energy NEXT intake*) OR circuit* OR ("high intensity" NEXT interval*) OR ("high intensity" NEXT intermittent*) OR "HIIT" OR (sprint NEXT interval*) OR (stair* NEAR/3 climbing) OR calisthenic* OR cycling* OR biking* OR [mh "Sedentary Behavior"] OR sedentary OR (physical NEXT inactivit*)):ti,ab,kw |
| #3 | Mortality & Recurrence | ([mh "Neoplasm Recurrence, Local"] OR [mh "Recurrence"] OR recurren* OR reoccurren* OR relaps* OR [mh "Neoplasms, Second Primary"] OR "secondary events" OR (second NEXT cancer*) OR ("second primary" NEXT cancer*) OR (secondary NEXT cancer*) OR ("secondary primary" NEXT cancer*) OR [mh "Disease Progression"] OR progression* OR [mh "Disease-Free Survival"] OR (cancer NEXT survivor*) OR (cancer NEAR/3 survival) OR (cause NEAR/3 death) OR "all cause" OR fatal* OR [mh "Mortality"] OR mortalit* OR [mh "Quality of Life"] OR "quality of life" OR "life quality" OR "HRQO"):ti,ab,kw |
| #4 |  | #1 AND #2 AND #3 |
| #5 | Limits & Filters | [mh "Editorial"] OR [mh "Comment"] OR commentary* OR [mh "News"] OR [mh "Letter"] OR [mh "Retracted publication"] OR [mh "Retraction of publication"] OR (retraction NEAR/2 publication*) OR [mh "Congress"] OR [mh "Consensus Development Conference"] OR (conference NEXT abstract*) OR (conference NEXT proceeding*) OR (conference NEXT paper*) OR (conference NEXT review*) OR symposium* OR [mh "Case Reports"] OR (case NEXT report*) OR [mh "Review"] OR [mh "Systematic Review"] OR [mh "Systematic Reviews as Topic"] OR "systematic review" OR [mh "Meta-Analysis"] OR [mh "Meta-Analysis as Topic"] OR "meta-analysis" OR "meta-analyses" OR [mh "Practice Guideline"] |
| #6 | Limits & Filters | ("retraction notice" OR "retracted publication" OR review OR protocol OR protocols OR guideline*):ti |
| #7 |  | (#5 OR #6) |
| #8 | Limits & Filters | #4 NOT #7" with Publication Year from 2003 to 2023, with Cochrane Library publication date Between Jan 2003 and Sep 2023, in Trials (Word variations have been searched) |

Database: Embase
Platform: Elsevier
Date Searched: 10/04/24

Date Limits: 1/1/2003 – 10/04/24

|  | Concept: | Search Strategy: |
| --- | --- | --- |
| #1 | Breast Cancer | 'breast cancer'/exp OR ((breast* NEAR/4 (cancer* OR neoplas* OR carcinoma* OR tumor* OR tumour* OR malignan* OR sarcoma* OR lymphoma*)):ab,ti) |
| #2 | Physical Activity | 'exercise'/exp OR 'kinesiotherapy'/exp OR 'fitness'/exp OR 'endurance'/exp OR 'recreation'/de OR 'sport'/exp OR 'tai chi'/exp OR 'walking'/exp OR 'sedentary lifestyle'/exp OR 'exercise*':ab,ti OR 'physical fitness':ab,ti OR 'physical activit*':ab,ti OR 'physical performanc*':ab,ti OR 'physical exertion':ab,ti OR ((physical NEAR/4 endurance):ab,ti) OR 'endurance training':ab,ti OR 'physical intensit*':ab,ti OR 'recreational activit*':ab,ti OR 'sport*':ab,ti OR 'stretching':ab,ti OR 'yoga':ab,ti OR 'tai ji':ab,ti OR 'tai chi':ab,ti OR ((muscle NEAR/4 strengthening):ab,ti) OR 'resistance training':ab,ti OR ((strength NEAR/4 training):ab,ti) OR 'muscle strength*':ab,ti OR 'weight training':ab,ti OR 'weightlifting':ab,ti OR 'weight lifting':ab,ti OR 'walk*':ab,ti OR 'gait':ab,ti OR 'jog':ab,ti OR 'jogging':ab,ti OR 'running':ab,ti OR 'swimming':ab,ti OR 'aerobic*':ab,ti OR 'exertion*':ab,ti OR 'isometric*':ab,ti OR 'energy expenditure*':ab,ti OR 'energy balanc*':ab,ti OR 'energy intake*':ab,ti OR 'circuit*':ab,ti OR 'high intensity interval*':ab,ti OR 'high intensity intermittent*':ab,ti OR 'hiit':ab,ti OR 'sprint interval*':ab,ti OR ((stair* NEAR/3 climbing):ab,ti) OR 'calisthenic*':ab,ti OR 'cycling*':ab,ti OR 'biking*':ab,ti OR 'sedentary':ab,ti OR 'physical inactivit*':ab,ti |
| #3 | Mortality & Recurrence | 'tumor recurrence'/exp OR 'recurrent disease'/exp OR 'second primary neoplasm'/exp OR 'disease exacerbation'/exp OR 'disease free survival'/exp OR 'mortality'/mj OR 'quality of life'/exp OR 'recurren*':ab,ti OR 'reoccurren*':ab,ti OR 'relaps*':ab,ti OR 'secondary events':ab,ti OR 'second cancer*':ab,ti OR 'second primary cancer*':ab,ti OR 'secondary cancer*':ab,ti OR 'secondary primary cancer*':ab,ti OR 'progression*':ab,ti OR 'cancer survivor*':ab,ti OR ((cancer NEAR/3 survival):ab,ti) OR ((cause NEAR/3 death):ab,ti) OR 'all cause':ab,ti OR 'fatal*':ab,ti OR 'mortalit*':ab,ti OR 'quality of life':ab,ti OR 'life quality':ab,ti OR 'hrqol':ab,ti |
| #4 | Limits & Filters | #1 AND #2 AND #3 AND ([article]/lim OR [article in press]/lim)  AND [2003-2023]/py AND [english]/lim NOT ([animals]/lim NOT ([animals]/lim AND [humans]/lim)) NOT ('editorial'/exp OR 'letter'/exp OR 'note'/exp OR 'abstract report'/exp OR 'conference paper'/exp OR 'review'/exp OR 'systematic review'/exp OR 'systematic review (topic)'/exp OR 'meta analysis'/exp OR 'meta analysis (topic)'/exp OR 'case report'/exp OR 'practice guideline'/exp OR 'retraction notice'/exp OR 'retraction of publication':ab,ti OR 'retraction notice':ti OR 'retracted publication':ab,ti OR 'systematic review':ab,ti OR 'meta analysis':ab,ti OR 'meta analyses':ab,ti OR 'review':ti OR 'protocol':ti OR 'protocols':ti OR 'guideline*':ti OR 'case report*':ab,ti OR [conference abstract]/lim OR [conference paper]/lim OR [conference review]/lim OR 'symposium*':ab,ti OR 'conference abstract*':ab,ti OR 'conference review*':ab,ti OR 'conference proceeding*':ab,ti OR 'conference paper*':ab,ti) |

Database: CINAHL Plus
Platform: EBSCO*host*
Date Searched: 10/04/24

Date Limits: 1/1/2003 – 10/04/24

|  | Concept: | Search Strategy: |
| --- | --- | --- |
| #S1 | Breast Cancer | (MH "Breast Neoplasms+") OR TI ( (((MH "Breast+") OR breast*) AND (cancer* OR neoplas* OR carcinoma* OR tumor* OR tumour* OR malignan* OR sarcoma* OR lymphoma*)) ) OR AB ( (((MH "Breast+") OR breast*) AND (cancer* OR neoplas* OR carcinoma* OR tumor* OR tumour* OR malignan* OR sarcoma* OR lymphoma*)) ) |
| #S2 | Physical Activity | ((MH "Exercise+") OR (MH "Therapeutic Exercise+") OR (MH "Physical Fitness+") OR (MH "Exertion+") OR (MH "Physical Endurance+") OR (MH "Recreation") OR (MH "Sports+") OR (MH "Yoga+") OR (MH "Tai Chi") OR (MH "Walking+") OR (MH "Life Style, Sedentary+") ) OR TI ( exercise* OR "physical fitness" OR "physical activit*" OR "physical performanc*" OR "physical exertion" OR (physical W4 endurance) OR "endurance training" OR "physical intensit*" OR "recreational activit*" OR "sport*" OR "stretching" OR "yoga" OR "Tai Ji" OR "Tai Chi" OR "muscle strength*" OR (muscle W4 strengthening") OR "resistance training" OR (strength W4 training") OR "muscle strength*" OR "weight training" OR "weightlifting" OR "weight lifting" OR "walk*" OR "gait" OR "jog" OR "jogging" OR "running" OR "swimming" OR "aerobic*" OR "exertion*" OR "isometric*" OR "energy expenditure*" OR "energy balanc*" OR "energy intake*" OR "circuit*" OR "high intensity interval*" OR "high intensity intermittent*" OR "HIIT" OR "sprint interval*" OR (stair* W3 climbing) OR calisthenic* OR cycling* OR "biking* OR sedentary OR "physical inactivit*" ) OR AB ( exercise* OR "physical fitness" OR "physical activit*" OR "physical performanc*" OR "physical exertion" OR (physical W4 endurance) OR "endurance training" OR "physical intensit*" OR "recreational activit*" OR "sport*" OR "stretching" OR "yoga" OR "Tai Ji" OR "Tai Chi" OR "muscle strength*" OR (muscle W4 strengthening") OR "resistance training" OR (strength W4 training") OR "muscle strength*" OR "weight training" OR "weightlifting" OR "weight lifting" OR "walk*" OR "gait" OR "jog" OR "jogging" OR "running" OR "swimming" OR "aerobic*" OR "exertion*" OR "isometric*" OR "energy expenditure*" OR "energy balanc*" OR "energy intake*" OR "circuit*" OR "high intensity interval*" OR "high intensity intermittent*" OR "HIIT" OR "sprint interval*" OR (stair* W3 climbing) OR calisthenic* OR cycling* OR "biking* OR sedentary OR "physical inactivit*") |
| #S3 | Mortality & Recurrence | ( (MH "Neoplasm Recurrence, Local") OR (MH "Recurrence+") OR (MH "Neoplasms, Second Primary") OR (MH "Disease Progression+") OR (MH "Disease-Free Survival") OR (MH "Mortality+") OR (MH "Quality of Life+") ) OR TI ( recurren* OR reoccurren* OR relaps* OR "secondary events" OR "second cancer*" OR "second primary cancer*" OR "secondary cancer*"OR "secondary primary cancer*" OR progression* OR "cancer survivor*" OR (cancer N/3 survival) OR (cause N/3 death) OR "all cause" OR fatal* OR mortalit* OR "quality of life" OR "life quality" OR "HRQOL" ) OR AB ( recurren* OR reoccurren* OR relaps* OR "secondary events" OR "second cancer*" OR "second primary cancer*" OR "secondary cancer*"OR "secondary primary cancer*" OR progression* OR "cancer survivor*" OR (cancer N/3 survival) OR (cause N/3 death) OR "all cause" OR fatal* OR mortalit* OR "quality of life" OR "life quality" OR "HRQOL" ) |
| #S4 | Limits & Filters | #S4 NOT ( (((MH "Animals+") OR (MH "Animal Studies") OR (TI "animal model*")) NOT (MH "human")) ) NOT ( (MH "Congresses and Conferences") OR (MH "Edit and Review+") OR (MH "News") OR (MH "Literature Review+") OR (MH "Meta Analysis") OR (MH "Systematic Review") OR (MH "Retracted Publication") OR (MH "Retraction of Publication) OR (MH "Case Studies") OR (MH "Practice Guidelines") ) OR ( "conference abstract*" OR "conference proceeding*" OR "conference paper*" OR "conference review*" OR "symposium*" OR "case report*" OR "systematic review" OR "meta-analysis" OR "meta-analyses" OR "retraction of publication*" ) OR TI ( review OR protocol OR protocols OR "retraction notice" OR "retracted publication" OR guideline* ) Limiters - Publication Year: 2003-2023; English Language; Peer Reviewed Expanders - Apply equivalent subjects Search modes - Boolean/Phrase |

Database: PsycInfo
Platform: American Psychological Association
Date Searched: 10/04/24

Date Limits: 1/1/2003 – 10/04/24

|  | Concept: | Search Strategy: |
| --- | --- | --- |
| #1 | Breast Cancer | Index Terms: {Breast Neoplasms} *OR* Title: (( OR breast*) AND (cancer* OR neoplas* OR carcinoma* OR tumor* OR tumour* OR malignan* OR sarcoma* OR lymphoma*)) *OR* Abstract: (( OR breast*) AND (cancer* OR neoplas* OR carcinoma* OR tumor* OR tumour* OR malignan* OR sarcoma* OR lymphoma*)) |
| #2 | Physical Activity | Index Terms:  OR {Exercise Therapy} OR {Physical Fitness} OR {Physical Endurance} OR {Recreation} OR OR {Yoga} OR {Walking} OR {Sedentary Behavior} *OR* Title: exercise* OR "physical fitness" OR "physical activit*" OR "physical performanc*" OR "physical exertion" OR (physical NEAR/4 endurance) OR "endurance training" OR "physical intensit*" OR "recreational activit*" OR "sport*" OR "stretching" OR "yoga" OR "Tai Ji" OR "Tai Chi" OR "muscle strength*" OR (muscle NEAR/4 strengthening") OR "resistance training" OR (strength NEAR/4 training") OR "muscle strength*" OR "weight training" OR "weightlifting" OR "weight lifting" OR "walk*" OR "gait" OR "jog" OR "jogging" OR "running" OR "swimming" OR "aerobic*" OR "exertion*" OR "isometric*" OR "energy expenditure*" OR "energy balanc*" OR "energy intake*" OR "circuit*" OR "high intensity interval*" OR "high intensity intermittent*" OR "HIIT" OR "sprint interval*" OR (stair* NEAR/3 climbing) OR calisthenic* OR cycling* OR "biking* OR sedentary OR "physical inactivit*" *OR* Abstract: exercise* OR "physical fitness" OR "physical activit*" OR "physical performanc*" OR "physical exertion" OR (physical NEAR/4 endurance) OR "endurance training" OR "physical intensit*" OR "recreational activit*" OR "sport*" OR "stretching" OR "yoga" OR "Tai Ji" OR "Tai Chi" OR "muscle strength*" OR (muscle NEAR/4 strengthening") OR "resistance training" OR (strength NEAR/4 training") OR "muscle strength*" OR "weight training" OR "weightlifting" OR "weight lifting" OR "walk*" OR "gait" OR "jog" OR "jogging" OR "running" OR "swimming" OR "aerobic*" OR "exertion*" OR "isometric*" OR "energy expenditure*" OR "energy balanc*" OR "energy intake*" OR "circuit*" OR "high intensity interval*" OR "high intensity intermittent*" OR "HIIT" OR "sprint interval*" OR (stair* NEAR/3 climbing) OR calisthenic* OR cycling* OR "biking* OR sedentary OR "physical inactivit*" |
| #3 | Mortality & Recurrence | Index Terms: {Relapse (Disorders)} OR {Disease Progression} OR {Death and Dying} OR {Quality of Life} OR Title: recurren* OR Title: reoccurren* OR Title: relaps* OR Title: "secondary events" OR Title: "second cancer*" OR Title: "second primary cancer*" OR Title: "secondary cancer*" OR "secondary primary cancer*" OR Title: progression* OR Title: "cancer survivor*" OR (Title: cancer NEAR/3 survival) OR (Title: cause NEAR/3 death) OR Title: "all cause" OR Title: fatal* OR Title: mortalit* OR Title: "quality of life" OR Title: "life quality" OR Title: "HRQOL" OR Abstract: recurren* OR Abstract: reoccurren* OR Abstract: relaps* OR Abstract: "secondary events" OR Abstract: "second cancer*" OR Abstract: "second primary cancer*" OR Abstract: "secondary cancer*" OR "secondary primary cancer*" OR Abstract: progression* OR Abstract: "cancer survivor*" OR (Abstract: cancer NEAR/3 survival) OR (Abstract: cause NEAR/3 death) OR Abstract: "all cause" OR Abstract: fatal* OR Abstract: mortalit* OR Abstract: "quality of life" OR Abstract: "life quality" OR Abstract: "HRQOL" |
| #4 | Limits & Filters | ((IndexTermsFilt: ("Breast Neoplasms")) OR (Any Field: TitleFilt: ((("Breast" OR breast*)) AND TitleFilt: ((cancer* OR neoplas* OR carcinoma* OR tumor* OR tumour* OR malignan* OR sarcoma* OR lymphoma*)))) OR (Any Field: AbstractFilt: ((("Breast" OR breast*)) AND AbstractFilt: ((cancer* OR neoplas* OR carcinoma* OR tumor* OR tumour* OR malignan* OR sarcoma* OR lymphoma*))))) AND ((IndexTermsFilt: ("Exercise") OR IndexTermsFilt: ("Exercise Therapy") OR IndexTermsFilt: ("Physical Fitness") OR IndexTermsFilt: ("Physical Endurance") OR IndexTermsFilt: ("Recreation") OR IndexTermsFilt: ("Sports") OR IndexTermsFilt: ("Yoga") OR IndexTermsFilt: ("Walking") OR IndexTermsFilt: ("Sedentary Behavior")) OR title: (exercise* OR "physical fitness" OR "physical activit*" OR "physical performanc*" OR "physical exertion" OR (physical NEAR/4 endurance) OR "endurance training" OR "physical intensit*" OR "recreational activit*" OR "sport*" OR "stretching" OR "yoga" OR "Tai Ji" OR "Tai Chi" OR "muscle strength*" OR (muscle NEAR/4 strengthening ") OR " resistance training " OR (strength NEAR/4 training") OR "muscle strength*" OR "weight training" OR "weightlifting" OR "weight lifting" OR "walk*" OR "gait" OR "jog" OR "jogging" OR "running" OR "swimming" OR "aerobic*" OR "exertion*" OR "isometric*" OR "energy expenditure*" OR "energy balanc*" OR "energy intake*" OR "circuit*" OR "high intensity interval*" OR "high intensity intermittent*" OR "HIIT" OR "sprint interval*" OR (stair* NEAR/3 climbing) OR calisthenic* OR cycling* OR "biking* OR sedentary OR " physical inactivit* ") OR Abstract:(exercise* OR " physical fitness " OR " physical activit* " OR " physical performanc* " OR " physical exertion " OR (physical NEAR/4 endurance) OR " endurance training " OR " physical intensit* " OR " recreational activit* " OR " sport* " OR " stretching " OR " yoga " OR " Tai Ji " OR " Tai Chi " OR " muscle strength* " OR (muscle NEAR/4 strengthening") OR Any Field: "resistance training" OR (strength NEAR/4 training ") OR " muscle strength* " OR " weight training " OR " weightlifting " OR " weight lifting " OR " walk* " OR " gait " OR " jog " OR " jogging " OR " running " OR " swimming " OR " aerobic* " OR " exertion* " OR " isometric* " OR " energy expenditure* " OR " energy balanc* " OR " energy intake* " OR " circuit* " OR " high intensity interval* " OR " high intensity intermittent* " OR " HIIT " OR " sprint interval* " OR (stair* NEAR/3 climbing) OR calisthenic* OR cycling* OR " biking* OR sedentary OR "physical inactivit*")) AND ((IndexTermsFilt: ("Relapse (Disorders)") OR IndexTermsFilt: ("Disease Progression") OR IndexTermsFilt: ("Death and Dying") OR IndexTermsFilt: ("Quality of Life")) OR (title: (recurren*) OR title: (reoccurren*) OR title: (relaps*) OR title: ("secondary events") OR title: ("second cancer*") OR title: ("second primary cancer*") OR title: ("secondary cancer*" OR "secondary primary cancer*") OR title: (progression*) OR title: ("cancer survivor*") OR (title: (cancer NEAR/3 survival)) OR (title: (cause NEAR/3 death)) OR title: ("all cause") OR title: (fatal*) OR title: (mortalit*) OR title: ("quality of life") OR title: ("life quality") OR title: ("HRQOL")) OR (abstract: (recurren*) OR abstract: (reoccurren*) OR abstract: (relaps*) OR abstract: ("secondary events") OR abstract: ("second cancer*") OR abstract: ("second primary cancer*") OR abstract: ("secondary cancer*" OR "secondary primary cancer*") OR abstract: (progression*) OR abstract: ("cancer survivor*") OR (abstract: (cancer NEAR/3 survival)) OR (abstract: (cause NEAR/3 death)) OR abstract: ("all cause") OR abstract: (fatal*) OR abstract: (mortalit*) OR abstract: ("quality of life") OR abstract: ("life quality") OR abstract: ("HRQOL"))) AND Language: English AND NOT Population Group: Animal NOT Document Type: Abstract Collection OR Column/Opinion OR Comment/Reply OR Dissertation OR Editorial OR Letter OR Retraction OR Review-Book AND Peer-Reviewed Journals only AND Year: 2003 To 2023 |

Database: Web of Science (Core Collection)
Platform: Clarivate Analytics
Date Searched: 10/04/24

Date Limits: 1/1/2003 – 10/04/24

|  | Concept: | Search Strategy: |
| --- | --- | --- |
| #1 | Breast Cancer | TS=(breast* AND (cancer* OR neoplas* OR carcinoma* OR tumor* OR tumour* OR malignan* OR sarcoma* OR lymphoma*)) |
| #2 | Physical Activity | TS=(exercise* OR "physical fitness" OR "physical activit*" OR "physical performanc*" OR "physical exertion" OR (physical* NEAR/4 endurance*) OR "endurance training" OR "physical intensit*" OR "recreational activit*" OR sport* OR stretching OR yoga OR "Tai Ji" OR "Tai Chi" OR "muscle strength*" OR (muscle NEAR/4 strengthening) OR "resistance training" OR (strength NEAR/4 training) OR "muscle strength*" OR "weight training" OR "weightlifting" OR "weight lifting" OR walk*OR gait OR jog OR jogging OR running OR swimming OR aerobic* OR exertion* OR isometric* OR "energy expenditure*" OR "energy balanc*" OR "energy intake*" OR circuit* OR "high intensity interval*" OR "high intensity intermittent*" OR HIIT OR "sprint interval*" OR (stair NEAR/3 climbing) OR calisthenic* OR cycling* OR biking* OR sedentary OR "physical inactivit*") |
| #3 | Mortality & Recurrence | TS=(recurren* OR reoccurren* OR relaps* OR "secondary events" OR "second cancer*" OR "second primary cancer*" OR "secondary cancer*" OR "secondary primary cancer*" OR "progression*" OR "cancer survivor*" OR (cancer NEAR/3 survival) OR (cause NEAR/3 death) OR "all cause" OR fatal* OR mortalit* OR "quality of life" OR "life quality" OR HRQOL) |
| #4 | Limits & Filters | #3 AND #2 AND #1 NOT TS=(Commentary OR "retraction of publication*" OR "conference abstract*" OR "conference proceeding*" OR "conference paper*" OR "conference review*" OR "symposium*" OR "case report*" OR "systematic review" OR "meta-analysis" OR "meta-analyses")) NOT TI=(review OR "retraction notice" OR "retracted publication" OR protocol OR protocols OR guideline*) and Preprint Citation Index (Exclude – Database) and Clinical Trial or Other or Review Article or Clinical Trial or Abstract or Meeting or Dissertation Thesis or Data Set or Editorial Material or Book or Unspecified or Patent or Case Report or Data Study or Letter or News or Correction or Reference Material or Retracted Publication or Data Paper or Biography (Exclude – Document Types) and Animals or Disease Models Animal or Mice Inbred Balb C or Mice Nude or Mice or Cell Movement (Exclude – MeSH Headings) and English (Languages) and Web of Science Core Collection (Database) \| Timespan: 2003-01-01 to 2023-09-18 (Publication Date) |

Database: Scopus
Platform: Elsevier

Date Searched: 10/04/24

Date Limits: 1/1/2003 – 10/04/24

|  | Concept: | Search Strategy: |
| --- | --- | --- |
| #1 | Breast Cancer | TITLE-ABS-KEY(breast* AND (cancer* OR neoplas* OR carcinoma* OR tumor* OR tumour* OR malignan* OR sarcoma* OR lymphoma*)) |
| #2 | Physical Activity | TITLE-ABS-KEY(exercise* OR "physical fitness" OR "physical activit*" OR "physical performanc*" OR "physical exertion" OR (physical* W/4 endurance*) OR "endurance training" OR "physical intensit*" OR "recreational activit*" OR sport* OR stretching OR yoga OR "Tai Ji" OR "Tai Chi" OR "muscle strength*" OR (muscle W/4 strengthening) OR "resistance training" OR (strength W/4 training) OR "muscle strength*" OR "weight training" OR "weightlifting" OR "weight lifting" OR walk*OR gait OR jog OR jogging OR running OR swimming OR aerobic* OR exertion* OR isometric* OR "energy expenditure*" OR "energy balanc*" OR "energy intake*" OR circuit* OR "high intensity interval*" OR "high intensity intermittent*" OR HIIT OR "sprint interval*" OR (stair W/3 climbing) OR calisthenic* OR cycling* OR biking* OR sedentary OR "physical inactivit*") |
| #3 | Mortality & Recurrence | TITLE-ABS-KEY(recurren* OR reoccurren* OR relaps* OR "secondary events" OR "second cancer*" OR "second primary cancer*" OR "secondary cancer*" OR "secondary primary cancer*" OR "progression*" OR "cancer survivor*" OR (cancer W/3 survival) OR (cause W/3 death) OR "all cause" OR fatal* OR mortalit* OR "quality of life" OR "life quality" OR HRQOL) |
| #4 | Limits & Filters | (TITLE-ABS-KEY((breast* AND (cancer* OR neoplas* OR carcinoma* OR tumor* OR tumour* OR malignan* OR sarcoma* OR lymphoma*)))) AND (TITLE-ABS-KEY(exercise* OR "physical fitness" OR "physical activit*" OR "physical performanc*" OR "physical exertion" OR (physical* W/4 endurance*) OR "endurance training" OR "physical intensit*" OR "recreational activit*" OR sport* OR stretching OR yoga OR "Tai Ji" OR "Tai Chi" OR "muscle strength*" OR (muscle W/4 strengthening) OR "resistance training" OR (strength W/4 training) OR "muscle strength*" OR "weight training" OR "weightlifting" OR "weight lifting" OR walk*OR gait OR jog OR jogging OR running OR swimming OR aerobic* OR exertion* OR isometric* OR "energy expenditure*" OR "energy balanc*" OR "energy intake*" OR circuit* OR "high intensity interval*" OR "high intensity intermittent*" OR HIIT OR "sprint interval*" OR (stair W/3 climbing) OR calisthenic* OR cycling* OR biking* OR sedentary OR "physical inactivit*")) AND (TITLE-ABS-KEY(recurren* OR reoccurren* OR relaps* OR "secondary events" OR "second cancer*" OR "second primary cancer*" OR "secondary cancer*" OR "secondary primary cancer*" OR "progression*" OR "cancer survivor*" OR (cancer W/3 survival) OR (cause W/3 death) OR "all cause" OR fatal* OR mortalit* OR "quality of life" OR "life quality" OR HRQOL)) AND NOT INDEXTERMS (animal OR animals) AND NOT ((TITLE-ABS-KEY(Commentary OR "retraction of publication*" OR "conference abstract*" OR "conference proceeding*" OR "conference paper*" OR "conference review*" OR "symposium*" OR "case report*" OR "systematic review" OR "meta-analysis" OR "meta-analyses") OR TITLE(review OR "retraction notice" OR "retracted publication" OR protocol OR protocols OR guideline*))) AND PUBYEAR > 2002 AND PUBYEAR < 2024 AND (EXCLUDE (DOCTYPE,"re") OR EXCLUDE (DOCTYPE,"ch") OR EXCLUDE (DOCTYPE,"no") OR EXCLUDE (DOCTYPE,"ed") OR EXCLUDE (DOCTYPE,"le") OR EXCLUDE (DOCTYPE,"cp") OR EXCLUDE (DOCTYPE,"bk") OR EXCLUDE (DOCTYPE,"cr")) |

**Supplement 2.** Inclusion and exclusion criteria

| **Criteria** | **Inclusion** | **Exclusion** |
| --- | --- | --- |
| Date | - Published 2003 to October 2024 | - Published prior to October 2024 |
| Population | - Breast cancer survivors | - Non-human subjects - Non-breast cancer survivors |
| Exposure | - Measure post-diagnosis recreational and/or leisure-time aerobic or muscle-strengthening activity and/or exercise | - Do not measure post-diagnosis activity (i.e. focus on pre-diagnosis exercise) - Measure total activity or other domains (e.g. occupational exercise), or focus specifically on sports - Combine aerobic and muscle-strengthening activity together into single variable - Use a poor-quality activity measure (e.g. any vs. none) |
| Outcome | - Quality of life | - The absence of quality of life as an outcome (happiness, life satisfaction, health status measures framed as quality of life were excluded) |
| Type of Evidence | - Primary observational empirical research studies (e.g., cohort studies and cross-sectional studies) available in full text | - Trials/interventions - Reviews and meta-analyses - Editorials (e.g., perspectives, commentaries) - Abstracts, conference proceedings or posters - Dissertations/theses - Research protocols - Case reports - Patents - Articles for which the full text cannot be obtained |
| Statistical Analysis | - Physical activity treated as the exposure and HRQOL treated as the outcome - Analysis accounted for covariates/confounders | - HRQOL treated as the exposure and physical activity treated as the outcome - Univariate analysis only |
| Language | - English | - Not available in English |

Notes. HRQOL = Health-Related Quality of Life;

**Supplement 3**. Minimally Important Differences (MID) for Physical, Emotional, Social, Global/General, and Breast Cancer–Specific HRQOL Domains

| Domain | FACT-B | MID | SF-36 | MID | EORTC-QLQ | MID | EQ-5D-3L | MID |
| --- | --- | --- | --- | --- | --- | --- | --- | --- |
| Physical well-being and function | Physical Well-Being (PWB) | 2 to 3 ^[64, 65]^ | Physical functioning | 2 to 4 ^[66]^ | Physical functioning | 2 to 7 ^[67]^ |  |  |
|  | Functional Well-Being (FWB) | 2 to 3 ^[64, 65]^ | Role functioning/physical | 2 to 4 ^[66]^ | Role functioning | 6 to 12 ^[67]^ |  |  |
|  | Trial Outcome Index (TOI) = PWB+FWB+BCS | 5 to 6 ^[68]^ | Energy/fatigue | 2 to 4 ^[66]^ | Fatigue | 4 to 9 ^[67]^ |  |  |
|  |  |  | Pain | 2 to 4 ^[66]^ | N/V | 3 to 9 ^[67]^ |  |  |
|  |  |  | Physical component summary | 2 ^[66]^ | Pain | 5 to 9 ^[67]^ |  |  |
|  |  |  |  |  | Dyspnea | 2 to 9 ^[67]^ |  |  |
|  |  |  |  |  | Insomnia | 5 to 9 ^[67]^ |  |  |
|  |  |  |  |  | Appetite loss | 7 to 13 ^[67]^ |  |  |
|  |  |  |  |  | Constipation | 4 to 10 ^[67]^ |  |  |
|  |  |  |  |  | Diarrhea | 3 to 11 ^[67]^ |  |  |
| Emotional well-being, function, and mental health | Emotional Well-Being (EWB) | 2 to 3 ^[64, 65]^ | Role functioning/emotional | 2 to 4 ^[66]^ | Emotional functioning | 6 to 9 ^[67]^ |  |  |
|  |  |  | Emotional well-being | 2 to 4 ^[66]^ | Cognitive functioning | 3 to 7 ^[67]^ |  |  |
|  |  |  | Mental component summary | 3 ^[66]^ |  |  |  |  |
| Social well-being and function | Social/Familial Well-Being (SWB) | 2 to 3 ^[65]^ | Social functioning | 2 to 4 ^[66]^ | Social functioning | 3 to 8 ^[67]^ |  |  |
| Global/General | Total | 7 to 8 ^[68]^ | Total (not recommended) |  | Global health status/QoL | 5 to 8 ^[67]^ | Visual Analogue Scale (VAS) | 8 to 12 ^[69]^ |
|  | Total (FACT-G) | 3 to 7 ^[68]^ | General health | 2 to 4 ^[66]^ |  |  | Index score | 0.044  (Specific to Canada) ^[70]^ |
|  |  |  | Health change | 2 to 4 ^[66]^ |  |  |  |  |
| Breast cancer subscale | Breast Cancer Subscale (BCS) | 2 to 3 ^[68]^ |  |  |  |  |  |  |
| Other |  |  |  |  | Financial difficulties | >3 ^[67]^ |  |  |

Notes. FACT-B = Functional Assessment of Cancer Therapy - Breast; SF-36 = 36-Item Short Form Health Survey; EORTC-QLQ = European Organization for Research and Treatment of Cancer Quality of Life Questionnaire; EQ-5D-3L = EuroQol 5-Dimension 3-Level; PWB = Physical Well-Being; FWB = Functional Well-Being; TOI = Trial Outcome Index; BCS = Breast Cancer Subscale; EWB = Emotional Well-Being; SWB = Social/Familial Well-Being; VAS = Visual Analogue Scale; FACT-G = Functional Assessment of Cancer Therapy – General; MID = Minimally Important Difference

**Supplement 4.** Participant race/ethnicity, education, and income

| **Author** | **Location (Data source)** | **Race and/or ethnicity** | | | | | **Education** | | | | **Income** |
| --- | --- | --- | --- | --- | --- | --- | --- | --- | --- | --- | --- |
|  |  | **Black** | **White** | **Hispanic** | **Asian** | **Other** | **<HS** | **HS/GED** | **Some College** | **≥ College** |  |
| Milne et al. 2007 | Australia (Western Australia Cancer Registry) | - | | | | | Primary: 6.1% | 57.5% | Trade certificate: 2.9%  Diploma/degree: 30.5%  Masters/PhD: 3.0% | | <$60,000: 61.4%  ≥$60,000: 17.0%  Missing: 10.9% |
| Vallance et al. 2012 | Canada (Alberta Cancer Registry) | - | | | | | 12.1% | 21.0% | 26.1% | College: 31.1%  Some graduate school: 2.4%  Graduate school: 7.3% | <$60,000: 50.2%  ≥$60,000: 49.7% |
| Peck et al. 2022 | Canada (EMBRACE-MRI 1) | - | | | | | - | | | | - |
| Chen et al. 2009 | China (SBCSS) | - | - | - | 100% Chinese | - | 50.0% | 35.4% | >High school: 14.5% | | (**¥**/month/ capita)  <1,000: 66.3%  ≥1,000: 33.7% |
| Vehmanen et al. 2022 | Finland, Israel, Italy, Portugal (BOUNCE) | - | - | - | - | - | 0-9 years: 4.5%  >9 years: 95.5% | | | | Very low/low: 23.8%  Average/high: 76.2% |
| Sagen et al. 2009 | Norway (Hospitals ) | - | | | | | - | | | | - |
| Blanchard et al. 2008 | U.S. -National  (ACS SCS-II) | 12.4% | 72.0% | 9.0% | - | - | ≤ HS: 36.0% | | Some college or technical/vocational school: 28.0% | College graduate: 15.0%  Postgraduate: 14.3% | <$40,000: 40.9%  ≥$40,000: 38.0% |
| Dibble et al. 2021 | U.S. - CT (UConn Health) | 1.9% | 94.3% | 7.6% | 1.9 | American Indian/Alaska Native: 1.9% | 1.9% | 8.2% | Some college or technical/vocational school: 20.8% | College grad:18.2%  Some grad school: 9.4%  Grad degree: 41.5% | - |
| Alfano et al. 2007 | U.S. - NM, WA (HEAL) | 0.2% | 80.6% | 15.1% | - | Other: 4.2% | 20.7% | | 34.5% | College grad: 22.6%  Grad school: 22.2% | - |
| Pakiz et al. 2016 | U.S. - AL, CA, CO, MO, (ENERGY trial) | 10.2% | 79.0% | 6.6% | 1.6% | 2.2% | Non-college graduate: 40.1% | | | College grad: 59.2% | - |
| Hart et al. 2018 | U.S. WI (WISC) | - | Primarily of European descent | - | - | - | 4.0% | 38.0% | 27.3% | 30.7% | <$50,000: 46.9%  ≥$ 50,000: 53.1% |

Notes. ACS SCS-II = American Cancer Society’s Study of Cancer Survivors-II; ENERGY = Exercise and Nutrition to Enhance Recovery and Good Health for You; LACE = Life After Cancer Epidemiology; LILAC = Life and Longevity After Cancer; NHIS = National Health Interview Survey; SBCSS = Shanghai Breast Cancer Survival Study; WHEL= Women's Healthy Eating and Living; TEAM = Tamoxifen Exemestane Adjuvant Multicenter; WISC = Wisconsin in Situ Cohort; SD= Standard deviation; HS: High school; GED = General Educational Development

**Supplement 5.** Participant clinical characteristics

| **Author** | **Location (Data source)** | **Study Design** | **Gender** | **Stage** | **Age** | **Treatment** | **Time since diagnosis** |
| --- | --- | --- | --- | --- | --- | --- | --- |
| Milne et al. 2007 | Australia (Western Australia Cancer Registry) | Prospective cohort (n=558) | Women diagnosed with BC no longer undergoing active treatment; no secondary cancers | I: 31.9%  II: 21.5%  III: 12.4%  IV: 2.7%  Unknown: 31.5% | M=59.0 (SD= 11.2)  <65 years: 69.2%  ≥65 years: 30.8% | CT: 43.0%  CT alone: 17.9%  CT + RT: 39.2%  HT: 74.0%  RT: 64.3%  RT alone: 42.9%  Surg: 98.7% | M=25.2 (SD=3.4) months |
| Vallance et al. 2012 | Canada (Alberta Cancer Registry) | Retrospective cohort (n=524) | Women diagnosed with BC who had completed adjuvant therapy except hormone therapy and lived in rural and small towns | I: 45.4%  II:39.7%  III: 7.7% | ≥18 years; M=62.4 (SD=11.1) years; At diagnosis M=56.2 (SD=11.4) | First-line treatment:  CT: 0.2%  HT: 0.2%  RT: 0.2%  Surg: 99.4  Second-line treatment  CT: 49.8%  HT: 20.2%  RT: 29.8%  Surg: 0.2  Third-line treatment  CT: 0.2%  HT: 51.1  RT: 48.5 | M=76.4 (SD=36.5) months |
| Peck et al. 2022 | Canada (EMBRACE-MRI 1) | Prospective cohort (n=88) | Women with HER2+ breast cancer receiving anthracyclines followed by trastuzumab  therapy | I: 9%  II: 61%  III: 30%  IV: 1% | M=51.4 (SD= 8.9) years | CT: Cumulative epirubicin equivalent dose, mg/m^2^: 309.0  RT: 86%  Heart radiation dose, cGy: 187.0 | NR |
| Chen et al. 2009 | China (SBCSS) | Prospective cohort (n=1829) | Women diagnosed with incident BC | 0-I: 36.4%  IIA: 34.1%  IIB: 18.8%  III-IV: 6.9%  Unknown: 1.7% | M=53.7 (SD= 10.2) years | CT: 91.9%  HT (Tamoxifen): 58.9%  IT: 13.3%  RT: 28.4%  Surg (Mastectomy): 95.1% | 6 months |
| Vehmanen et al. 2022 | Finland, Israel, Italy, Portugal (BOUNCE) | Prospective cohort (n=311) | Women diagnosed with BC receiving adjuvant or neo-adjuvant therapy in addition to surgery | I: 47.3%  II: 43.7%  III: 9.0% | 40-70 years; M=55.41 (SD=7.99) | CT: 54.7%  HT: Endocrine treatment (84.6%),  Anti-HER2 (18.3%)  RT: 79.4%  Surg: Lumpectomy: (74.3%), Mastectomy: (25.7%)  Psychotropic medication: 27%  Psychological support: 11.6% | NR |
| Sagen et al. 2009 | Norway (Hospitals) | Prospective cohort (n=204) | Women diagnosed with early-stage BC who underwent mastectomy or breast-conserving surgery with axillary node dissection | Stages I-II: Details not reported | Aged 32-75 years; M=55 (SD=10) years | CT: 39%  HT: 52%  RT (supraclavicular nodes): 43%  Surg: Breast ablation (52%), Breast preservation (48%), Surgery at dominant side (53%) | NR |
| Blanchard, Courneya, & Stein, 2008 | U.S. - National (American Cancer Society’s Study of Cancer  Survivors-II (ACS SCS-II) | Cross-sectional (n=2885) | Women diagnosed BC in the calendar year either 2, 5, or 10 years before sampling | In situ: 0%  Local: 70.3%  Regional: 28.8%  Distant: 0.9% | M=63.2 (SD= 12.2) years | CT: 58.9%  HT: 12.9%  IT: 61.0%  RT: 62.8%  Surg: 95.1%  BMT: 13.2 | 2 years: 34.2%  5 years: 35.8%  10 years: 29.9% |
| Dibble et al. 2021 | U.S. - CT (UConn Health) | Cross-sectional cohort (n=170) | Postmenopausal women diagnosed with BC who had completed treatment | I: 49.5%  II: 33.6%  III: 5.6%  Atypical ductal hyperplasia: 0.9%  Ductal CIS: 9.3%  Lobular CIS: 9.3% | 50-95 years; M=68.7 (SD= 10.3) years | CT only: 1.2%  HT (AI): 60.3%  RT only: 1.2%  Surg: Lumpectomy only (0.6%),  Mastectomy only (1.2%),  Combination therapies without AT: 25.3% | M=10.1 (SD=6.6) years |
| Alfano et al. 2007 | U.S. - NM, WA (HEAL) | Prospective cohort (n=545) | Women diagnosed with BC | In situ: 23.5%  Local: 58.9%  Regional: 17.6% | <50: 20.9%  ≥50: 79.1%  M = 58.0 (SD = 10.3) years | Surg only: 29.0%  Surg +RT: 42.8%  Surg + CT: 7.0%  Surg +RT + CT: 21.3% | Diagnosis to baseline: M = 6.15 (SD = 1.84)  Diagnosis to follow-up: M = 29.35 (SD = 2.73)  Diagnosis to QOL questionnaire: M = 39.47 (SD = 6.31) |
| Pakiz et al. 2016 | U.S. - AL, CA, CO, MO (ENERGY trial) | Prospective cohort (n=692) | Women diagnosed with BC who were overweight or obese and had completed initial therapies (excluding HT) | I: 30.3%  II: 51.7%  III: 17.9% | M=56 (SD= 9) years | CT: 76.2%  HT: Antiestrogen only (21.2%),  Antiestrogen inhibitor (52.8%) | <1 year: 11.0%  1-2.9 years: 47.0%  ≥3 years: 42.0% |
| Hart et al. 2018 | U.S. - WI (WISC) | Prospective cohort (n=1448) | Women diagnosed with ductal carcinoma in situ | - | At diagnosis:  <55: 49.1%  ≥55: 50.9% | Surg:  Mastectomy (ipsilateral or bilatera;; 41.7%),  Breast conserving surgery without RT: 8.6%  Breast conserving surgery with RT: 47.3%  Biopsy only: 2.4 | M=1.3 (range: 0.3-4.0) years |

Notes. ENERGY = Exercise and Nutrition to Enhance Recovery and Good Health for You; LACE = Life After Cancer Epidemiology; LILAC = Life and Longevity After Cancer;  NHIS = National Health Interview Survey; SBCSS = Shanghai Breast Cancer Survival Study; WHEL= Women's Healthy Eating and Living; TEAM = Tamoxifen Exemestane Adjuvant Multicenter; WISC = Wisconsin in Situ Cohort; BMT= Bone marrow transplantation; BCT= Breast conserving therapy; CIS = Carcinoma In Situ; M = Mean; SD= standard deviation

**Supplement 6.** Recreational physical activity measurement and guidelines

| **Authors** | **Location (Data source)** | **Measure** | **Guidelines** | **Proportion meeting guidelines (%)** |
| --- | --- | --- | --- | --- |
| Milne et al. 2007 | Australia (Western Australia Cancer Registry) | GLTEQ | ≥60min/week of strenuous PA or ≥150min/week of MPA (Australian Public Health PA guidelines – uncited) | 31.0 |
| Vallance et al. 2012 | Canada (Alberta Cancer Registry) | GLTEQ | ≥150 min/week of MPA or ≥60 min/week of VPA (AHA/ACSM) | 34.7 |
| Peck et al. 2022 | Canada (EMBRACE-MRI 1) | GLTEQ | ≥90 min/week of MVPA (Cancer PA Guidelines) | 48.0 |
| Chen et al. 2009 | China (SBCSS) | SWHS PAQ (leisure-time) | ≥8.3 MET-hrs/week (USDHHS) | 6 months = 55.0  36 months (weighted) = 66.1 |
| Vehmanen et al. 2022 | Finland, Israel, Italy, Portugal (BOUNCE) | MPA and VPA (exercise) | ≥150min/week of PA (USDHHS) | 44.7 |
| Sagen et al. 2009 | Norway (Hospitals ) | Unnamed (leisure-time) | ≥150 min/week of MPA or ≥60 min/week of VPA (ACSM) | - |
| Blanchard et al. 2008 | U.S. -National  (ACS SCS-II) | GLTEQ | ≥150 min/week of MPA or ≥60 min/week of VPA (ACS) | 37.1 |
| Dibble et al. 2021 | U.S. - CT (UConn Health) | NHANES items (recreational) | ≥150-300min/week of MPA, or ≥75min/week of VPA/week or equivalent combination (ACSM)  ≥2 days/week of MSE | 50.0  26.5 MSE |
| Alfano et al. | U.S. - NM, WA (HEAL) | MAQ (sports/recreational) | - | - |
| Pakiz et al. 2016 | U.S. - AL, CA, CO, MO, (ENERGY trial) | GLTEQ | - | - |
| Hart et al. 2018 | U.S. - WI (WISC) | NHS items (recreational) | Regular participation ≥30 min/week and for ≥3 months/year | - |

Notes. ACS SCS-II = American Cancer Society’s Study of Cancer Survivors-II; ENERGY = Exercise and Nutrition to Enhance Recovery and Good Health for You; LACE = Life After Cancer Epidemiology; LILAC = Life and Longevity After Cancer; NHIS = National Health Interview Survey; SBCSS = Shanghai Breast Cancer Survival Study; WHEL= Women's Healthy Eating and Living; TEAM = Tamoxifen Exemestane Adjuvant Multicenter; WISC = Wisconsin in Situ Cohort; GLETQ = Godin Leisure Time Exercise Questionnaire; NHS = Nurses’ Health Study; MAQ = Modifiable Activity Questionnaire; NHANES = National Health and Nutrition Examination Survey; SWHS PAQ = Shanghai Women’s Health Study Physical Activity Questionnaire; Physical activity = Physical activity; MPA = Moderate intensity physical activity; VPA = Vigorous intensity physical activity; MVPA = Moderate-to-vigorous intensity physical activity; ACS = American Cancer Society; ACSM = American College of Sports Medicine; AHA = American Heart Association; USDHHS = US Department of Health and Human Services; MSE = Muscle-strengthening Exercise

**Supplement 7.** Risk of Bias Assessment

Studies generally performed well on confounding (Domain 1), with four out of eleven scoring "low risk." We pre-specified important confounders to be age at diagnosis, treatment type, stage at diagnosis, estrogen receptor/progesterone receptor hormone status, and comorbidities, which were largely controlled for, though estrogen receptor/progesterone receptor status was omitted in all studies except for Chen et al. Some studies adjusted for post-exposure variables affected by the exposure (e.g., body mass index), which can lead to over-adjustment bias [43, 46-48].

All studies were at "some concerns" for exposure measurement (Domain 2) due to the self-reported nature of leisure-time physical activity, which may be prone to measurement error [71].

Regarding participant selection and analysis (Domain 3), seven studies were classified as “high risk”. This classification was due to dropouts or non-responses, particularly when participants dropped out due to health reasons, which could be influenced by the exposure and outcome. Addressing dropouts or exclusions with sensitivity analyses improved the risk of bias for those studies that did so.

Four studies were at "very high risk" for missing data (Domain 5), as they did not meet the threshold of ≥80% of those recruited. Missing data could bias the results, especially when related to health status or quality of life. Again, studies reporting sensitivity analyses comparing those with missing data to those without missing data were rated at lower risk of bias compared to those studies that did not.

All studies were at “high risk” of bias for outcome measurement (Domain 6), as HRQOL outcomes were self-reported, which may have been influenced by participants’ knowledge of their physical activity levels.

Reporting bias was significant, with six out of eleven studies at “high risk” and five out of eleven at "some concerns." Most studies did not report or explain alternative analysis forms tested (e.g., continuous vs. categorical exposures and outcomes), which limited transparency. Additionally, several studies only reported selected HRQOL domains, which could introduce selective reporting bias.


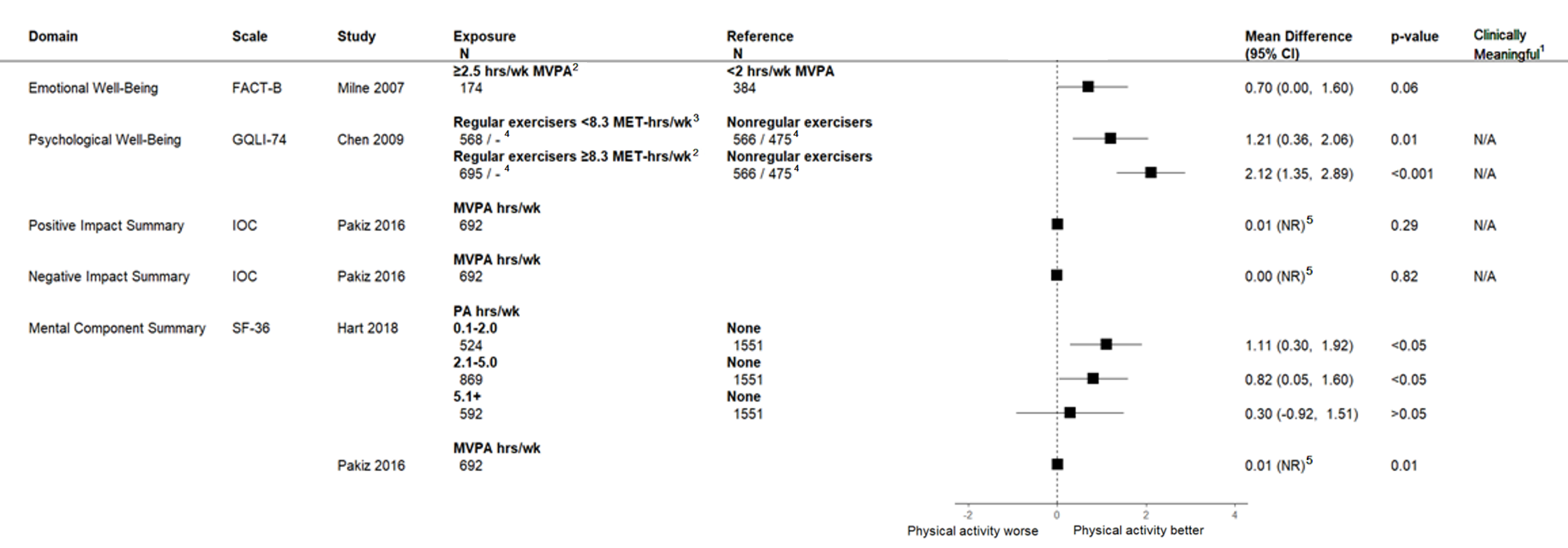


**Supplement 8**. Comparison of adjusted mean differences of emotional well-being, function, and mental health domains of HRQOL between breast cancer survivors by physical activity level (exposure vs. reference).

^1^Clinically meaningful was defined as a mean difference exceeding the minimally important difference threshold (see Supplement 1) and being statistically significant

^2^Corresponds to meeting aerobic activity guidelines

^3^Regular exercisers defined as individuals reporting exercising at least twice per week, while non-regular exercisers were those reporting exercising less than twice per week

^4^Results are derived from a mixed model. Sample sizes are reported for the 6-month and 36-month follow-up periods

^5^Results derived from a linear regression of a log-transformed outcome

Notes. HRQOL = Health-Related Quality of Life; CI = Confidence Interval; FACT-B = Functional Assessment of Cancer Therapy - Breast; SF-36 = 36-Item Short Form Health Survey; GQLI-74 = Generic Quality of Life Inventory - 74; EORTC QLQ-C30 = European Organization for Research and Treatment of Cancer Quality of Life Questionnaire - Core 30; MVPA = Moderate-to-Vigorous intensity Physical Activity; MET-hrs/wk = Metabolic Equivalent of Task Hours per Week; PA = Physical Activity; MSE = Muscle-strengthening Exercise; IOC = Impact of Cancer Scale; NR = Not Reported


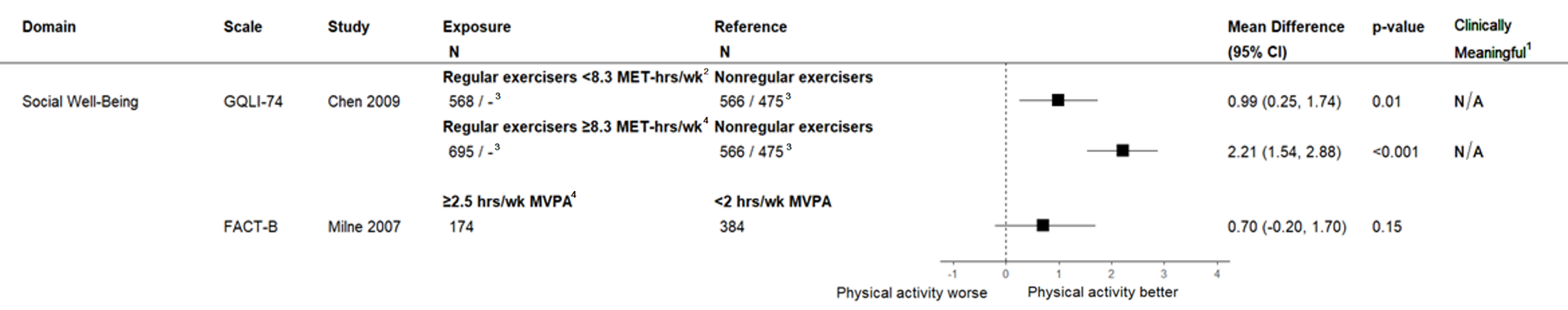


**Supplement 9**. Comparison of adjusted mean differences of social well-being domain of HRQOL between breast cancer survivors by physical activity level (exposure vs. reference).

^1^Clinically meaningful was defined as a mean difference exceeding the minimally important difference threshold (see Supplement 1) and being statistically significant.

^2^Regular exercisers defined as individuals reporting exercising at least twice per week, while non-regular exercisers were those reporting exercising less than twice per week

^3^Results are derived from a mixed model. Sample sizes are reported for the 6-month and 36-month follow-up periods

^4^Corresponds to meeting aerobic activity guidelines

Notes. HRQOL = Health-Related Quality of Life; CI = Confidence Interval; FACT-B = Functional Assessment of Cancer Therapy - Breast; GQLI-74 = Generic Quality of Life Inventory - 74; MVPA = Moderate-to-Vigorous intensity Physical Activity; MET-hrs/wk = Metabolic Equivalent of Task Hours per Week


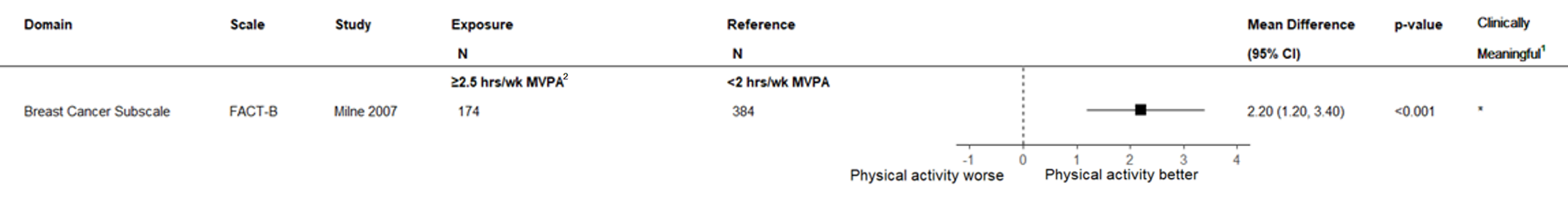


**Supplement 10**. Comparison of adjusted mean differences of breast cancer subscale domain of HRQOL between breast cancer survivors by physical activity level (exposure vs. reference).

^1^Clinically meaningful was defined as a mean difference exceeding the minimally important difference threshold (see Supplement 1) and being statistically significant.

^2^Corresponds to meeting aerobic activity guidelines

Notes. HRQOL = Health-Related Quality of Life; CI = Confidence Interval; FACT-B = Functional Assessment of Cancer Therapy - Breast; MVPA = Moderate-to-Vigorous intensity Physical Activity; MET-hrs/wk = Metabolic Equivalent of Task Hours per Week

**Supplement 11.** Summary of the association between physical activity and Global, Total, and General Health HRQOL Domains among breast cancer survivors

| Domain | Scale | | Study | | | Country | Target Population | HRQOL timing | | PA timing | | PA definition | Mean difference | | | | 95% CI | p-value |
| --- | --- | --- | --- | --- | --- | --- | --- | --- | --- | --- | --- | --- | --- | --- | --- | --- | --- | --- |
| Total/Global | | FACT-B | | Dibble et al. 2021 | U.S. | | Postmenopausal women diagnosed with stage 0-3a BC who completed treatment | | Post-treatment | | | Meeting MSE guidelines of ≥2 days/wk (<2 days/wk Ref) | 7.2 | | | -1.72, 16.12 | | >0.05 |
|  |  |  |  |  |  |  |  |  |  | | | Meeting MVPA guidelines of ≥2.5 hrs/wk (<2.5 hrs/wk Ref) | | 6.36 | | -3.50, 16.22 | | >0.05 |
|  |  |  |  | Milne et al. 2007 | Australia | | Women >18 years diagnosed with BC no longer undergoing active treatment and with no secondary cancers | | Post-treatment | | | Meeting MVPA guidelines of ≥2.5 hrs/wk (<2.5 hrs/wk Ref) | 8.4 | | | 4.8, 11.9 | | <0.001^a^ |
|  |  | FACT-G | | Dibble et al. 2021 | U.S. | | Postmenopausal women diagnosed with stage 0-3a BC who completed treatment | | Post-treatment | | | Meeting MVPA guidelines of ≥2.5 hrs/wk (<2.5 hrs/wk Ref) | 5.94 | | | -1.35, 13.23 | | >0.05 |
|  |  |  |  | Vallance et al. 2012 | Canada | | Women ≥18 years living in rural and small towns diagnosed with BC who completed adjuvant therapy except hormone therapy | | Post-treatment | | | Meeting MVPA guidelines of ≥2.5 hrs/wk post-treatment (<2.5 hrs/wk Ref) | 4.20 | | | 1.50, 6.90 | | <0.01 |
|  |  |  |  |  |  |  |  |  | Post-treatment | | On-treatment | Meeting MVPA guidelines of ≥2.5 hrs/wk post-treatment (<2.5 hrs/wk Ref) | | 6.90 | 3.80, 10.00 | | | 0.002 |
|  |  |  |  | Milne et al. 2007 | Australia | | Women >18 years diagnosed with BC no longer undergoing active treatment and with no secondary cancers | | Post-treatment | | | Meeting MVPA guidelines of ≥2.5 hrs/wk (<2.5 hrs/wk Ref) | 6.30 | | | 3.50, 9.00 | | <0.001^a^ |
|  |  | GQLI-74 | | Chen et al. 2009 | China | | Women aged 20-75 years with incident BC | | 0-6 months and 18-36 months post-diagnosis | | | Nonregular exercisers | Ref | | |  | |  |
|  |  |  |  |  |  |  |  |  |  |  |  | Regular exercisers <8.3 MET-hrs/wk | 0.95 | | | 0.35, 1.54 | | <0.01 |
|  |  |  |  |  |  |  |  |  |  |  |  | Regular exercisers ≥8.3 MET-hrs/wk | 1.79 | | | 1.25, 2.33 | | <0.001 |
|  |  | EORTC QLQ-C30 | | Vehmanen et al. 2022 | Finland, Israel, Italy, Portugal | | Women aged 40-70 years who received adjuvant or neo-adjuvant therapy in addition to surgery for stage I-III breast cancer | | 12-months after beginning adjuvant therapy | | | Inactive | Ref | | |  | | <0.01 |
|  |  |  |  |  |  |  |  |  |  |  |  | Fairly active | 10.77 | | | 4.91, 16.63 | |  |
|  |  |  |  |  |  |  |  |  |  |  |  | Active | 12.07 | | | 7.85, 16.29 | |  |
| Total/Global | | SF-36 | | Blanchard et al. 2008 | U.S. | | Women ≥18 years diagnosed with local, regional, or distant BC in the calendar year either 2, 5, or 10 years before sampling | | Long-term post-diagnosis | | | Meeting MVPA guidelines of ≥2.5 hrs/wk (<2.5 hrs/wk Ref) | 4.10 | | | 3.24, 4.96 | |  |
|  |  | EQ-5D-3L | | Peck et al. 2022 | Canada | | Women with HER2+ breast cancer receiving anthracyclines followed by trastuzumab  therapy | | On-treatment | | | ≥90 minutes MVPA/wk (<90 minutes MVPA/wk Ref) | 0.05 | | | 0.02, 0.09 | | 0.001 |
|  |  |  |  |  |  |  |  |  |  |  |  | MVPA minutes/wk (every 30 minute increase) | 0.003 | | | 0.001, 0.005 | | 0.008 |
| General Health | | SF-36 | | Alfano et al. 2007 | U.S. | | Women diagnosed with in-situ or stages I-IIIA BC | | Post-diagnosis | | | MET-hrs/wk (every 10 MET-hrs/wk increase) | 0.39 | | | -0.25, 1.03 | | >0.01^a^ |
|  |  | EQ-5D-3L | | Peck et al. 2022 | Canada | | Women with HER2+ breast cancer receiving anthracyclines followed by trastuzumab  therapy | | On-treatment | | | ≥90 minutes MVPA/wk  (<90 minutes MVPA/wk Ref) | 8.22 | | | 4.53, 11.91 | | <0.001 |
|  |  |  |  |  |  |  |  |  |  |  |  | MVPA minutes/wk (every 30 minute increase) | 0.43 | | | 0.15, 0.71 | | <0.01 |

^a^Results are from unadjusted MANOVA; per authors, MANCOVA results adjusting for covariates were similar, but not reported.

Notes. HRQOL = Health-Related Quality of Life; PA = Physical Activity; FACT-B = Functional Assessment of Cancer Therapy - Breast; FACT-G = Functional Assessment of Cancer Therapy - General; GQLI-74 = Generic Quality of Life Index - 74-item version; EORTC QLQ-C30 = European Organization for Research and Treatment of Cancer Quality of Life Questionnaire - Core 30; MET-hrs/wk = Metabolic Equivalent of Task Hours per Week; MVPA = Moderate-to-Vigorous Physical Activity; MPA = Moderate intensity Physical Activity; SF-36 = 36-Item Short Form Health Survey; EQ-5D-3L = EuroQoL 5-Dimension 3-Level; CI = Confidence Interval; BC = Breast Cancer; HER2+ = Human Epidermal Growth Factor Receptor 2

**Supplement 12.** Summary of the association between physical activity and Physical Well-being and Function Domains of HRQOL among breast cancer survivors

| Domain | Scale | Study | Country | Target Population | HRQOL timing | PA timing | PA definition | Mean difference | 95% CI | p-value |
| --- | --- | --- | --- | --- | --- | --- | --- | --- | --- | --- |
| Physical Well-Being | GQLI-74 | Chen et al. 2009 | China | Women aged 20-75 years with incident BC | 0-6 months and 18-36 months post-diagnosis | | Nonregular exercisers | Ref |  |  |
|  |  |  |  |  |  |  | Regular exercisers <8.3 MET-hrs/wk | 1.19 | 0.39, 2.00 | 0.004 |
|  |  |  |  |  |  |  | Regular exercisers ≥8.3 MET-hrs/wk | 2.01 | 1.28, 2.75 | <0.001 |
|  | FACT-B | Milne et al. 2007 | Australia | Women >18 years diagnosed with no longer undergoing active treatment and with no secondary cancers | Post-treatment | | Meeting MVPA guidelines of ≥2.5 hrs/wk (<2.5 hrs/wk Ref) | 1.90 | 1.10, 2.80 | <0.001^a^ |
| Physical Functioning | SF-36 | Alfano et al. 2007 | U.S. | Women diagnosed with in-situ or stages I-IIIA BC | Post-diagnosis | | MET-hrs/wk (every 10 MET-hrs/wk increase) | 1.73 | 1.06, 2.40 | <0.001 |
|  | EORTC QLQ-C30 | Sagen et al. 2009 | Norway | Women <75 years diagnosed with early-stage BC who underwent mastectomy or breast-conserving surgery with axillary node dissection | 5-years post-surgery | 6-months post-surgery | PA min/wk | 0.08 | 0.04, 0.11 | <0.001 |
| Functional Well-Being | FACT-B | Dibble et al. 2021 | U.S. | Postmenopausal women diagnosed with stage 0-3a BC who completed treatment | Post-treatment | | Meeting MVPA guidelines of ≥2.5 hrs/wk (<2.5 hrs/wk Ref) | 2.77 | -0.56, 6.10 | >0.05 |
|  |  | Milne et al. 2007 | Australia | Women >18 years diagnosed with BC no longer undergoing active treatment and with no secondary cancers | Post-treatment | | Meeting MVPA guidelines of ≥2.5 hrs/wk (<2.5 hrs/wk Ref) | 2.90 | 1.90, 3.90 | <0.001^a^ |
| Role Functioning | SF-36 | Alfano et al. 2007 | U.S. | Women diagnosed with in-situ or stages I-IIIA BC | Post-diagnosis | | MET-hrs/wk (every 10 MET-hrs/wk increase) | -0.07 | -1.27, 1.12 | >0.01 |
| Trial Outcome Index | FACT-B | Dibble et al. 2021 | U.S. | Postmenopausal women diagnosed with stage 0-3a BC who completed treatment | Post-treatment | | Meeting MSE guidelines of ≥2 days/wk (<2 days/wk Ref) | 4.74 | -1.61, 11.09 | >0.05 |
|  |  | Milne et al. 2007 | Australia | Women >18 years diagnosed with BC no longer undergoing active treatment and with no secondary cancers | Post-treatment | | Meeting MVPA guidelines of ≥2.5 hrs/wk (<2.5 hrs/wk Ref) | 7.10 | 4.60, 9.60 | <0.001^a^ |
| Trial Outcome Index - Fatigue | FACT-G | Vallance et al. 2012 | Canada | Women ≥18 years living in rural and small towns diagnosed with BC who completed adjuvant therapy except hormone therapy | Post-treatment | | Meeting MVPA guidelines of ≥2.5 hrs/wk post-treatment (<2.5 hrs/wk Ref) | 6.90 | 3.80, 10.00 | 0.000 |
|  |  |  |  |  | Post-treatment | On-treatment | Meeting MVPA guidelines of ≥2.5 hrs/wk post-treatment (<2.5 hrs/wk Ref) | 7.90 | 3.60, 12.20 | 0.000 |
| Physical Component Summary | SF-36 | Hart et al. 2018 | U.S. | Women diagnosed with ductal carcinoma in situ, aged 20-74 years at diagnosis | Post-diagnosis | | PA hrs/wk |  |  |  |
|  |  |  |  |  |  |  | None | Ref |  |  |
|  |  |  |  |  |  |  | 0.1-2.0 | 1.06 | 0.25, 1.88 | <0.05 |
|  |  |  |  |  |  |  | 2.1-7.0 | 0.99 | 0.19, 1.80 | <0.05 |
|  |  |  |  |  |  |  | 7.1+ | 1.86 | 0.72, 3.20 | <0.05 |
|  |  | Pakiz et al. 2016 | U.S. | Overweight or obese women ≥21 years diagnosed with stage I-III BC within the previous 5 years and completed initial therapies (excluding endocrine therapy) | Post-treatment | | MVPA hrs/wk | 0.01 | NR | 0.006^b^ |
| *Symptoms* |  |  |  |  |  |  |  |  |  |  |
| Pain | SF-36 | Alfano et al. 2007 | U.S. | Women diagnosed with in-situ or stages I-IIIA BC | Post-diagnosis | | MET-hrs/wk (every 10 MET-hrs/wk increase) | 0.06 | -0.70, 0.81 | >0.01 |

^a^Results are from unadjusted MANOVA; per authors, MANCOVA results adjusting for covariates were similar, but not reported.

^b^Outcome was log-transformed.

Notes. HRQOL = Health-Related Quality of Life; PA = Physical Activity; GQLI-74 = Generic Quality of Life Index - 74-item version; FACT-B = Functional Assessment of Cancer Therapy - Breast; SF-36 = 36-Item Short Form Health Survey; EORTC QLQ-C30 = European Organization for Research and Treatment of Cancer Quality of Life Questionnaire - Core 30; EQ-5D-3L = EuroQol 5-Dimension 3-Level; MET-hrs/wk = Metabolic Equivalent of Task Hours per Week; MVPA = Moderate-to-Vigorous intensity Physical Activity; MSE = Muscle-strengthening Exercise; FACT-G = Functional Assessment of Cancer Therapy – General; CI = Confidence Interval; BC = Breast Cancer

**Supplement 13.** Summary of the association between physical activity and Emotional Well-being, Function, and Mental Health Domains of HRQOL among breast cancer survivors

| Domain | Scale | Study | Country | Target Population | HRQOL timing | PA timing | PA definition | Mean difference | 95% CI | p-value |
| --- | --- | --- | --- | --- | --- | --- | --- | --- | --- | --- |
| Emotional Well-Being | FACT-B | Milne et al. 2007 | Australia | Women >18 years diagnosed with BC in 2002 no longer undergoing active treatment and with no secondary cancers | Post-treatment | | Meeting MVPA guidelines of ≥2.5 hrs/wk (<2.5 hrs/wk Ref) | 0.70 | 0.00, 1.60 | 0.06^a^ |
| Psychological Well-Being | GQLI-74 | Chen et al. 2009 | China | Women aged 20-75 years with incident BC | 0-6 months and 18-36 months post-diagnosis | | Nonregular exercisers | Ref |  |  |
|  |  |  |  |  |  |  | Regular exercisers <8.3 MET-hrs/wk | 1.21 | 0.36, 2.06 | <0.01 |
|  |  |  |  |  |  |  | Regular exercisers ≥8.3 MET-hrs/wk | 2.12 | 1.35, 2.89 | <0.001 |
| Positive Impact Summary | IOC | Pakiz et al. 2016 | U.S. | Overweight or obese women ≥21 years diagnosed with stage I-III BC within the previous 5 years and completed initial therapies (excluding endocrine therapy) | Post-treatment | | MVPA hrs/wk | 0.01 | NR | 0.29^b^ |
| Negative Impact Summary | IOC | Pakiz et al. 2016 | U.S. | Overweight or obese women ≥21 years diagnosed with stage I-III BC within the previous 5 years and completed initial therapies (excluding endocrine therapy) | Post-treatment | | MVPA hrs/wk | -0.002 | NR | 0.82^b^ |
| Mental Component Summary | SF-36 | Hart et al. 2018 | U.S. | Women diagnosed with ductal carcinoma in situ, aged 20-74 years at diagnosis | Post-diagnosis | | PA hrs/wk |  |  |  |
|  |  |  |  |  |  |  | None | Ref |  |  |
|  |  |  |  |  |  |  | 0.1-2.0 | 1.11 | 0.30, 1.92 | <0.05 |
|  |  |  |  |  |  |  | 2.1-7.0 | 0.82 | 0.05, 1.60 | <0.05 |
|  |  |  |  |  |  |  | >7.1 | 0.30 | -0.92, 1.51 | >0.05 |
|  |  | Pakiz et al. 2016 | U.S. | Overweight or obese women ≥21 years diagnosed with stage I-III BC within the previous 5 years and completed initial therapies (excluding endocrine therapy) | Post-treatment | | MVPA hrs/wk | 0.01 | NR | 0.01^b^ |

^a^Results are from unadjusted MANOVA; per authors, MANCOVA results adjusting for covariates were similar, but not reported.

^b^Outcome was log-transformed.

Notes. HRQOL = Health-Related Quality of Life; PA = Physical Activity; FACT-B = Functional Assessment of Cancer Therapy - Breast; GQLI-74 = Generic Quality of Life Index - 74-item version; IOC = Impact of Cancer; SF-36 = 36-Item Short Form Health Survey; MET-hrs/wk = Metabolic Equivalent of Task Hours per Week; MVPA = Moderate-to-Vigorous intensity Physical Activity; CI = Confidence Interval; BC = Breast Cancer

**Supplement 14.** Summary of the association between physical activity and Social Well-being Domain of HRQOL among breast cancer survivors

| Domain | Scale | Study | Country | Target Population | HRQOL timing | PA timing | PA definition | Mean difference | 95% CI | p-value |
| --- | --- | --- | --- | --- | --- | --- | --- | --- | --- | --- |
| Social Well-Being | GQLI-74 | Chen et al. 2009 | China | Women aged 20-75 years with incident BC | 0-6 months and 18-36 months post-diagnosis | | Nonregular exercisers |  |  |  |
|  |  |  |  |  |  |  | Regular exercisers <8.3 MET-hrs/wk | 0.99 | 0.25, 1.74 | 0.01 |
|  |  |  |  |  |  |  | Regular exercisers ≥8.3 MET-hrs/wk | 2.21 | 1.54, 2.88 | <0.001 |
|  | FACT-B | Milne et al. 2007 | Australia | Women >18 years diagnosed with BC no longer undergoing active treatment and with no secondary cancers | Post-treatment | | Meeting MVPA guidelines of ≥2.5 hrs/wk (<2.5 hrs/wk Ref) | 0.7 | -0.2, 1.7 | 0.15^a^ |

^a^Results are from unadjusted MANOVA; per authors, MANCOVA results adjusting for covariates were similar, but not reported.

Notes. HRQOL = Health-Related Quality of Life; PA = Physical Activity; FACT-B = Functional Assessment of Cancer Therapy - Breast; GQLI-74 = Generic Quality of Life Index - 74-item version; MET-hrs/wk = Metabolic Equivalent of Task Hours per Week; MVPA = Moderate-to-Vigorous intensity Physical Activity; CI = Confidence Interval; BC = Breast Cancer

**Supplement 15.** Summary of the association between physical activity and Breast Cancer–Specific HRQOL Domain among breast cancer survivors

| Domain | Scale | Study | Country | Target Population | HRQOL timing | PA timing | PA definition | Mean difference | 95% CI | p-value |
| --- | --- | --- | --- | --- | --- | --- | --- | --- | --- | --- |
| Breast Cancer Subscale | FACT-B | Milne et al. 2007 | Australia | Women >18 years diagnosed with BC no longer undergoing active treatment and with no secondary cancers | Post-treatment | | Meeting MVPA guidelines of ≥2.5 hrs/wk (<2.5 hrs/wk Ref) | 2.20 | 1.20, 3.40 | <0.001^a^ |

^a^Results are from unadjusted MANOVA; per authors, MANCOVA results adjusting for covariates were similar, but not reported.

Notes. HRQOL=Health-related quality of life; FACT-B = Functional Assessment of Cancer Therapy – Breast; BC = Breast Cancer; CI = Confidence Interval; PA = Physical Activity; MVPA = Moderate-to-Vigorous intensity Physical Activity

**Supplement 16.** Grading of Recommendations Assessment, Development and Evaluation (GRADE) of Certainty of Evidence

| **Certainty assessment** | | | | | | | | **Sample Size** | **Effect** | | **Certainty** | |  |
| --- | --- | --- | --- | --- | --- | --- | --- | --- | --- | --- | --- | --- | --- |
| **№ of studies** | **Study design** | **Risk of bias** | **Inconsistency** | **Indirectness** | **Imprecision** | **Other considerations** | |  | **Mean Difference (95% CI)** | |  |  |  |
| **Physical Well-Being** | | | | | | |  | | | | |  |  |
| 2 | non-randomised studies | very serious^a^ | not serious | not serious | serious^b^ | none | | 2,387 | Studies showed small improvement in PWB not meeting threshold for minimally important difference (MID). | | ⨁◯◯◯  Very low | |  |
| **Physical Functioning** | | | | | | |  | | | | |  |  |
| 2 | non-randomised studies | very serious^a^ | not serious | not serious | serious^b^ | none | | 702 | Studies showed small improvement in PF not meeting threshold for minimally important difference (MID). | | ⨁◯◯◯  Very low | |  |
| **Functional Well-Being** | | | | | | |  | | | | |  |  |
| 2 | non-randomised studies | very serious^a^ | not serious | not serious | serious^b^ | none | | 679 | Studies showed small improvement in FWB not meeting threshold for minimally important difference (MID). | | ⨁◯◯◯  Very low | |  |
| **Role Functioning** | | | | | | |  | | |  | |  |  |
| 1 | non-randomised studies | very serious^a^ | not serious | not serious | not serious | none | | 545 | MD **0.07 points lower**  (1.27 lower to 1.12 higher) | | ⨁⨁◯◯  Low^b^ | |  |
| **Trial Outcome Index (TOI)** | | | | | | |  | | | | |  |  |
| 2 | non-randomised studies | very serious^a^ | not serious | not serious | serious^b^ | none | | 679 | Studies showed improvements in TOI, with one meeting the threshold for minimally important difference (MID). | | ⨁◯◯◯  Very low | |  |
| **TOI - Fatigue** | | | | | | |  | | | | |  |  |
| 1 | non-randomised studies | not serious | not serious | not serious | serious^b^ | none | | 514 | On-treatment MD **7.90 points higher**  (3.60 higher to 12.20 higher)  Post-treatment MD **6.90 points higher**  (3.80 higher to 10.00 higher) | | ⨁⨁⨁◯  Moderate | |  |
| **Physical Component Summary** | | | | | | |  | | | | |  |  |
| 2 | non-randomised studies | very serious^a^ | not serious | not serious | not serious | none | | 4,228 | Studies showed small improvement in PCS not meeting threshold for minimally important difference (MID). | | ⨁⨁◯◯  Low | |  |
| **Pain** | | | | | | |  | | | | |  |  |
| 1 | non-randomised studies | very serious^a^ | not serious | not serious | not serious | none | | 545 | MD **0.06 points higher**  (0.7 lower to 0.81 higher) | | ⨁⨁◯◯  Low | |  |
| **Total/Global** | | | | | | |  | | | | |  |  |
| 6 | non-randomised studies | very serious^a^ | not serious | not serious | serious^b^ | none | | 6,812 | Studies showed improvement in total/global HRQOL, with 3 meeting threshold for minimally important difference (MID). | | ⨁◯◯◯  Very low | |  |
| **General Health** | | | | | | |  | | | | |  |  |
| 1 | non-randomised studies | very serious^a^ | not serious | not serious | serious^b^ | none | | 545 | MD **0.39 points higher**  (0.25 lower to 1.03 higher) | | ⨁◯◯◯  Very low | |  |

**CI:** confidence interval; **MD:** mean difference

#### **Explanations**

a. See risk of bias evaluation

b. Imprecision in relation to minimally important difference (MID) and/or not meeting optimal information size per GRADE criteria

c. In relation to MID threshold

**Supplement 17**. Comparison of randomized controlled trial and observational study findings on the association between physical activity and general health perceptions among breast cancer survivors

| Randomized Controlled Trial |  | Observational |
| --- | --- | --- |
| Weighted mean difference (95% CI) |  | Adjusted mean difference (95% CI) |
| **SF-36** | | |
| - Aerobic^a^: 0.21 (-2.11, 2.52)  - Muscle-strengthening^a^: 4.66 (-0.24 to 9.56) |  | - Aerobic^b^: 0.39 (-0.25, 1.03)  - Muscle-strengthening: NR^c^ |

^a^Estimate associated with physical activity intervention.

^b^Estimate associated with every 10 metabolic equivalent of task (MET)-hours/week increase.

^c^No estimate reported for muscle-strengthening exercise.

Notes. SF-36 = 36-Item Short Form Survey

**Supplement 18**. Comparison of randomized controlled trial and observational study findings on the association between physical activity and physical function health-related quality of life among breast cancer survivors

| Randomized Controlled Trial |  | Observational |
| --- | --- | --- |
| Weighted mean difference (95% CI) |  | Adjusted mean difference (95% CI) |
| **SF-36** | | |
| - Aerobic^a^: 1.41 (-0.68, 3.50)  - Muscle-strengthening^a^: 2.84 (-2.00, 7.68) |  | - Aerobic^b^: 1.73 (1.06, 2.40)  - Muscle-strengthening: NR^c^ |
| **EORTC QLQ-C30** | | |
| - Aerobic^a^: 2.35 (-3.96, 8.67)  - Muscle-strengthening^a^: 4.78 (1.89, 7.67) |  | - Aerobic^d^: 0.08 (0.04, 0.11)  - Muscle-strengthening: NR^c^ |

^a^Estimate associated with physical activity intervention.

^b^Estimate associated with every 10 metabolic equivalent of task (MET)-hour/week increase.

^c^No estimate reported for muscle-strengthening exercise.

^d^Estimate associated with minutes/week of physical activity

Notes. SF-36 = 36-Item Short Form Survey; EROTC QLQ-C30 = European Organisation for Research and Treatment of Cancer Quality of Life Questionnaire C30

.

**Supplement 19**. Comparison of randomized controlled trial and observational study findings on the association between physical activity and physical component score (PCS) among breast cancer survivors

| Randomized Controlled Trial |  | Observational |
| --- | --- | --- |
| Weighted mean difference (95% CI) |  | Adjusted mean difference (95% CI) |
| **SF-36** | | |
| - Aerobic^a^: 0.30 (-1.29, 1.89)  - Muscle-strengthening^a^: 1.56 (-0.48, 3.60) |  | - Aerobic^b^: 1.06-1.86 (0.19, 3.20)  - Muscle-strengthening: NR^c^ |

^a^Estimate associated with physical activity intervention.

^b^Range of estimates for 0.1-2.0, 2.1-5.0, 5.1+ hours/week of physical activity compared to 0 hours/week of physical activity.

^c^No estimate reported for muscle-strengthening exercise.

Notes. SF-36 = 36-Item Short Form Survey

**Supplement 20**. Comparison of randomized controlled trial and observational study findings on the association between physical activity and mental component score (MCS) among breast cancer survivors

| Randomized Controlled Trial |  | Observational |
| --- | --- | --- |
| Weighted mean difference (95% CI) |  | Adjusted mean difference (95% CI) |
| **SF-36** | | |
| - Aerobic^a^: 2.98 (0.86, 5.10)  - Muscle-strengthening^a^: 0.59 (-3.41, 4.60) |  | - Aerobic^b^: 0.30-1.11 (-0.92, 1.92)  - Muscle-strengthening: NR^c^ |

^a^Estimate associated with physical activity intervention.

^b^Range of estimates for 0.1-2.0, 2.1-5.0, 5.1+ hours/week of physical activity compared to 0 hours/week of physical activity.

^c^No estimate reported for muscle-strengthening exercise.
